# Inductive‐Associative Meta‐learning Pipeline with Human Cognitive Patterns for Unseen Drug‐Target Interaction Prediction

**DOI:** 10.1002/advs.202506404

**Published:** 2025-07-01

**Authors:** Xiaoqing Lian, Tianxu Lv, Jie Zhu, Shiyun Nie, Hang Fan, Guosheng Wu, Yunjun Ge, Hong Xu, Xiaoting Wang, Lihua Li, Xiangxiang Zeng, Xiang Pan

**Affiliations:** ^1^ School of Artificial Intelligence and Computer Science Jiangnan University Wuxi Jiangsu 214122 China; ^2^ Department of Basic Medical Science, Wuxi School of Medicine Jiangnan University Wuxi Jiangsu 214122 China; ^3^ Department of Oncology The Changshu Affiliated Hospital of Soochow University Suzhou Jiangsu 215500 China; ^4^ Jiangnan University Medical Center (Wuxi No. 2 People's Hospital) Wuxi 214000 China; ^5^ Institute of Intelligent Biomedicine Hangzhou Dianzi University Hangzhou Zhejiang 310018 China; ^6^ College of Information Science and Engineering Hunan University Changsha Hunan 410082 China; ^7^ The PRC Ministry of Education Engineering Research Center of Intelligent Technology for Healthcare Wuxi Jiangsu 214122 China

**Keywords:** drug‐target interaction, inductive‐associative, meta‐learning, virtual screening

## Abstract

Significant differences in protein structures hinder the generalization of existing drug‐target interaction (DTI) models, which often rely heavily on pre‐learned binding principles or detailed annotations. In contrast, BioBridge designs an Inductive‐Associative pipeline inspired by the workflow of scientists who base their accumulated expertise on drawing insights into novel drug‐target pairs from weakly related references. BioBridge predicts novel drug–target interactions using limited sequence data, incorporating multi‐level encoders with adversarial training to accumulate transferable binding principles. On these principles basis, BioBridge employs a dynamic prototype meta‐learning framework to associate insights from weakly related annotations, enabling robust predictions for previously unseen drug‐target pairs. Extensive experiments demonstrate that BioBridge surpasses existing models, especially for unseen proteins. Notably, when only homologous protein binding data is available, BioBridge demonstrates robust efficacy for virtual screening of the epidermal growth factor receptor and adenosine receptor, underscoring its potential in drug discovery.

## Graphical abstract

Predicting unseen drug‐target interactions is challenging. BioBridge presents an Inductive‐Associative pipeline inspired by scientists' workflow. It combines transferable binding principles, learned via multi‐level encoders and adversarial training, with insights from weakly related references through meta‐learning. This enables robust DTI predictions for novel proteins, even with limited sequence information, offering new potential for drug discovery.

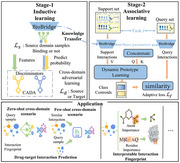

## 1 Introduction

Identifying drug‐target interactions (DTIs) is a cornerstone of drug discovery and development, significantly influencing various biological processes.^[^ ^1^ ^]^ Traditional methods rely heavily on labor‐intensive and fund‐intensive experimental analyses of chemical compounds,^[^ ^2^, ^3^ ^]^ which are increasingly complemented by computational approaches.^[^ ^4^, ^5^, ^6^ ^]^ Current computational methods include regression models predicting interaction strengths (e.g., Ki, IC50) and classification models identifying binding interactions based on potency thresholds.^[^ ^4^, ^7^ ^]^

DTI prediction methods typically fall into two categories: inductive and analogical. Inductive models utilize representations such as 3D structures or 2D sequences^[^ ^8^ ^]^ and employ advanced architectures like graph neural networks to generate embeddings for drug‐protein pairs.^[^ ^9^, ^10^, ^11^, ^12^, ^13^, ^14^ ^]^ Although effective under controlled conditions, these models often fail to generalize to novel drug–target pairs, as they primarily memorize existing annotations rather than identifying universal interaction mechanisms.^[^ ^15^, ^16^ ^]^ Analogical methods focus on discovering correlations using fine‐grained interaction annotations related to the drug‐target pairs of interest.^[^ ^17^, ^18^ ^]^ Despite their promise, these methods require highly specific data, making the development of new drugs challenging. A key limitation in both approaches is the significant variation among proteins, which hampers the transferability of learned drug‐target binding mechanisms to novel interactions.

This fundamental challenge mirrors scientists' cognitive barriers in drug development, motivating our integration of inductive and associative strategies that emulate human reasoning workflows. Inductive reasoning involves understanding and applying established criteria for drug‐target interactions, while associative strategies include consulting relevant literature to inform the process. This dual approach underscores the multifaceted nature of drug discovery, which extends beyond the mere application of existing knowledge or reliance on external data sources.^[^ ^19^, ^20^ ^]^

Drawing inspiration from this workflow, we propose BioBridge, an inductive‐associative pipeline designed to predict novel drug‐target interactions with high accuracy and minimal cost. BioBridge operates in two stages: induction and association. In the induction phase, a multi‐scale perception encoder identifies binding patterns across different levels, while adversarial learning strategies filter transferable binding principles. In the association phase, BioBridge mimics the literature review process by employing clustering‐based task partitioning and designs a dynamic prototypical meta‐learning algorithm to infer reliable interactions from limited binding annotations.

In a proof‐of‐concept study, we conducted extensive experiments on cold‐pair, cross‐domain zero‐shot, and few‐shot split, showing that BioBridge consistently outperforms other methods. Particularly for novel drug‐target pairs, BioBridge demonstrates a significant enhancement of up to 30% over inductive methods by including transferable interaction mechanisms and extracting insights from sparse, weakly correlated interaction annotations. Interaction predictions of the epidermal growth factor and adenosine receptor families further validate BioBridge's reliability, given the limited and inconsistent protein‐drug data. Ablation studies and interpretability analyses also offer valuable insights for future research.

## 2 Results

### 2.1 BioBridge Pipeline

**Figure 1:**
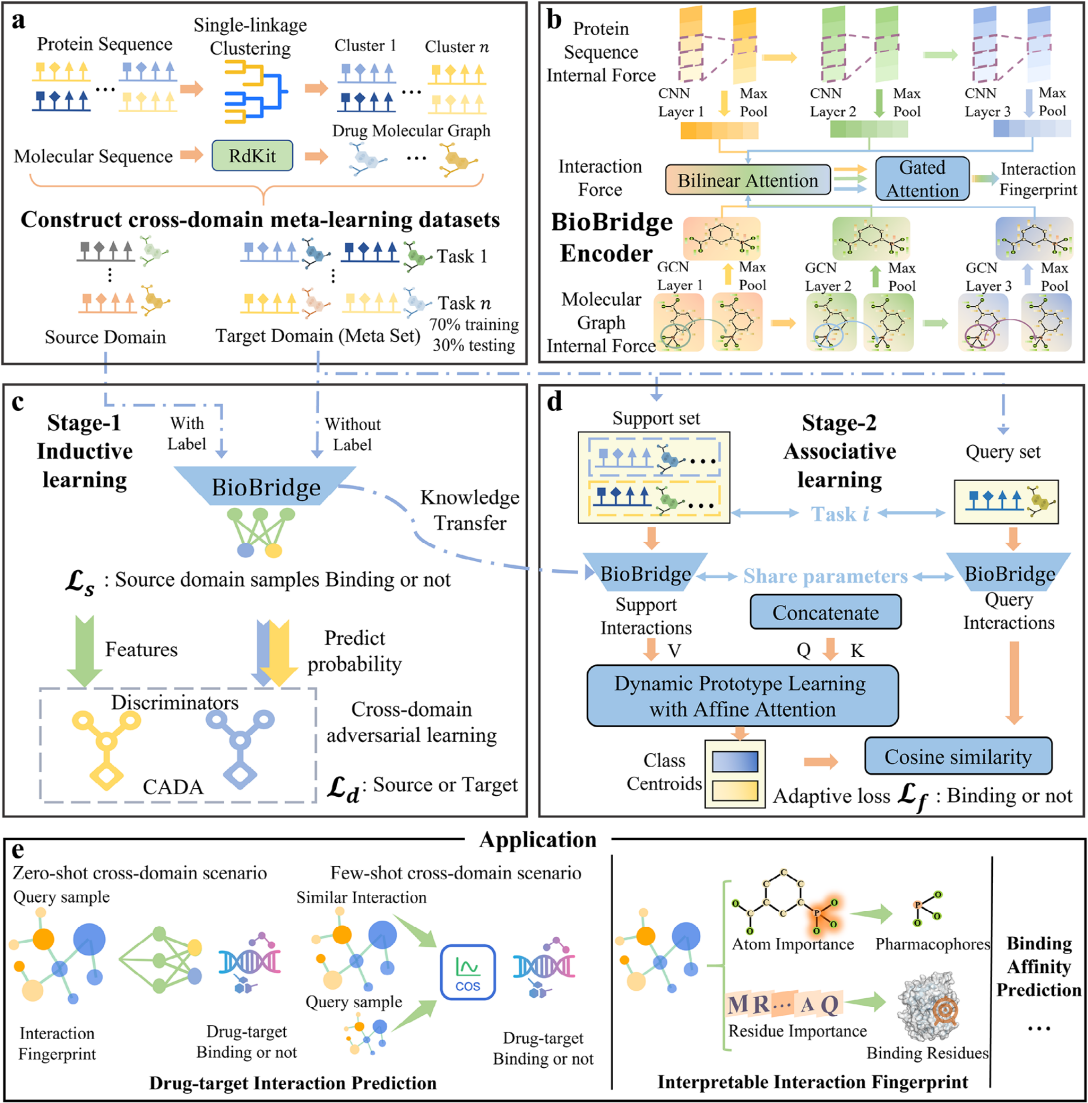
a) Dataset Preparation: Protein sequences are clustered into n classes. Drug molecular sequences are represented as molecular graphs via the RdKit.^[^ ^21^ ^]^ Based on protein clusters, data is split into source and target domains, then divided into meta‐tasks. b) BioBridge Encoder: BioBridge inputs protein sequences and molecular graphs using CNN and GCN to model internal forces. Bilinear attention captures interactions at multiple levels, while gated attention aggregates interpretable interaction fingerprints. c) Stage One: BioBridge pre‐trains with labeled source data and unlabeled target data. The loss function $L_{s}$ learns binding information, while $L_{d}$ handles cross‐domain adversarial learning. This binding knowledge is transferred to the next stage. d) Stage Two: Tasks from the target domain, form support and query sets. The concatenated interactions are treated as Q and K, and support interactions as V. A dynamic prototype learning module defines unique class prototypes. Cosine similarity determines binding status, with an adaptive loss function $L_{f}$ facilitating learning. e) BioBridge generalizes well across tasks, providing interpretable interaction fingerprints for biological insights.

BioBridge predicts DTIs by leveraging protein sequences and molecular graphs derived from drug sequences (**Figure** **1**a). It trains on annotated data, including binding affinities and functional effects, framing interaction predictions as binary classifications. For validation, proteins are clustered into known (source) and unknown (target) domains, with the target domain split into training and testing sets based on clustering within the meta‐learning setup. This cross‐domain configuration mimics the data distribution in real‐world drug development, and the meta‐tasks are designed to emulate the process scientists follow when reviewing relevant literature. Section S6 (Supporting Information) details how we remove data redundancy.

BioBridge emphasizes the importance of multi‐level understanding in drug target prediction, noting that shallow model features are more transferable to novel domains.^[^ ^22^, ^23^ ^]^ This motivates the use of multi‐level protein and molecular features to identify universal binding patterns (Figure 1b). BioBridge captures intra‐molecular and intra‐protein forces through three convolutional layers,^[^ ^24^ ^]^ using max‐pooling to extract fundamental atoms and residues, and simulates ligand‐protein interactions with Bilinear Attention at each level, consolidating interactions through a gated mechanism.

BioBridge employs cross‐domain adversarial learning to synchronize positive and negative interaction information between known and unknown pairs, capturing transferable interaction mechanisms for unknown drug‐target pairs during the inductive‐learning stage (Figure 1c). These patterns are transferred to the meta‐learning model through parameter sharing. BioBridge differentiates between positive and negative interactions during the adversarial phase to avoid confusion.

BioBridge forecasts novel drug‐target interactions by leveraging pre‐learned binding principles and drawing inspiration from weakly correlated annotations of non‐consistent interactions in the meta‐learning phase (Figure 1d). Using a siamese network, BioBridge creates interaction prototypes for positive and negative interactions, categorizing new queries by cosine similarity. Predictions are refined using an affine attention‐based dynamic prototype algorithm and an adaptive loss function.

As depicted in Figure 1e, BioBridge predicts interactions for unknown drug‐target pairs in zero‐shot and few‐shot learning scenarios, providing biological interpretability through attention‐based visualization. Additionally, BioBridge is adept at predicting drug‐target binding affinities, enhancing the understanding of interactions between drugs and their targets.

For clarification of model notations during training, refer to Table S2 (Supporting Information). Further details of the experimental setup are provided in Section S3 (Supporting Information).

### 2.2 BioBridge Induces Transferable Interaction Principles

Zero‐shot prediction of drug‐target interactions is a pivotal evaluation task in modern drug discovery, as it measures a model's ability to capture established principles of drug‐target binding and generalize to unseen scenarios. To assess the inductive capabilities of BioBridge, we conducted experiments on three prominent datasets: BindingDB,^[^ ^30^, ^31^ ^]^ BioSNAP,^[^ ^12^, ^32^ ^]^ and Human.^[^ ^33^ ^]^ Recognizing the importance of addressing real‐world challenges in novel drug development, we adopted both cold‐pair splits (Cold) and cross‐domain splits (Cluster) to simulate unknown scenarios. For the cross‐domain splits, BioBridge utilized the Category‐Aware Domain Adversarial Learning (CADA) module to enhance its predictive accuracy.

**Figure 2:**
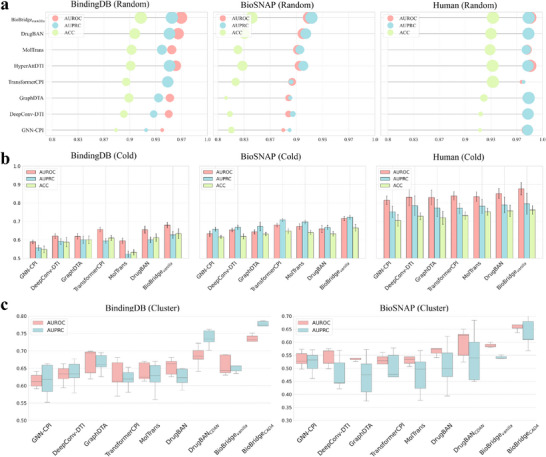
a) Mean performance comparison on the BindingDB, BioSNAP, and Human datasets using random splitting. The bubble size corresponds to the value of every metric. b) Comparison of cross‐domain performance on the BindingDB and BioSNAP datasets using cluster‐based pair splitting. Data are expressed as mean ± standard deviation. c) Zero‐shot comparison of meta cross‐domain splitting on BindingDB and BioSNAP datasets, indicating that protein differences are important factors limiting drug target prediction. Data are expressed as mean ± standard deviation.

As shown in **Figure** **2**a,b and Table S5 (Supporting Information), BioBridge consistently outperformed state‐of‐the‐art models that prioritize deep feature extraction, particularly in predicting interactions for unseen pairs. However, BioBridge's performance on random splits was less pronounced, indicating that while deep features effectively model known drug‐target interactions, shallow features demonstrate superior inductive capabilities for addressing unknown interaction mechanisms.

In cross‐domain evaluations, we incorporated the CADA module, forming BioBridge_*CADA*_. For a fair comparison, DrugBAN_*CDAN*_,^[^ ^25^, ^34^ ^]^ optimized for cross‐domain tasks, was also included. Due to the limited size of the Human dataset, experiments focused on the BindingDB and BioSNAP datasets, employing cluster‐based splitting^[^ ^25^ ^]^ to simulate cross‐domain scenarios. As illustrated in Figure 2c, performance declined across all models when evaluated on entirely novel drug‐target pairs. Despite this challenge, BioBridge demonstrated robust performance, surpassing non‐cross‐domain models on both datasets.

BioBridge_*CADA*_ achieved the highest results by effectively distinguishing domain differences and capturing transferable interaction patterns. This highlights the strength of combining a multi‐level encoder with the CADA module in establishing transferable principles of drug‐target interactions.

To further validate our approach, we compared BioBridge against structure‐based protein‐ligand models on the PDB2020 dataset.^[^ ^10^, ^35^ ^]^ Table S6 (Supporting Information) shows that BioBridge achieves leading results among sequence‐based models and is comparable to structure‐based models,^[^ ^36^, ^37^, ^38^, ^39^, ^40^, ^41^ ^]^ underscoring its adaptability and effectiveness even in data‐limited settings.

### 2.3 BioBridge Derives Inspiration by Association from Weakly Correlated Annotations

BioBridge inherits the transferable interaction principles learned during the inductive stage. To emulate the scientific process of drawing insights from references, we use meta‐learning to activate BioBridge's associative potential. Our first objective is to quantitatively identify the factors contributing to prediction errors for novel drug‐target pairs, which will guide the focus of subsequent experiments. To this end, we conducted evaluations under two distinct scenarios: cross‐domain novel drugs (Meta Unseen Drug) and cross‐domain novel proteins (Meta Unseen Protein). As shown in Figure S2 (Supporting Information), predicting interactions for entirely unknown proteins proved more challenging than for novel molecules, with performance differing by nearly 20%. This highlights a critical challenge in drug‐target prediction: the variability among proteins. Consequently, our few‐shot experiments focus on unseen proteins, employing BioBridge_*CADA*_'s final epoch parameters to ensure effective knowledge transfer.

Few‐shot learning assesses a model's ability to generalize when each class contains only *n* samples. Due to the limited size of the Human dataset, we focused on associative learning using the BindingDB and BioSNAP datasets. To derive insights from weakly related reference annotations for the target drug‐target pairs, we designed few‐shot tasks based on clustering target categories rather than individual classes (Section 4). By integrating the dynamic prototype learning module with the BioBridge encoder, we developed BioBridge_*Meta*_. For comparison, we also trained DrugBAN on both source and target training domain datasets and evaluated it on the target test domain.

Across both datasets, BioBridge_*Meta*_ achieved optimal or near‐optimal results on all metrics, with AUROC improvements of up to 30% in BindingDB, significantly outperforming inductive baseline methods (**Table** **1**). Notably, DrugBAN performed worse when trained on the target domain training set than when trained solely on the source domain, illustrating the shortcut learning tendency of inductive methods, which often memorize annotations instead of learning intrinsic interactions.^[^ ^25^ ^]^ The superior performance of BioBridge_*Meta*_ stems from its dynamic prototype algorithm, which effectively addresses protein differences and extracts meaningful information from weakly related reference annotations. This adaptability also explains the strong performance of MetaOptNet, which incorporates mechanisms for adaptive prototype construction. Additionally, in tasks where the support and query sets involved the same protein, BioBridge demonstrates remarkable performance, as shown in Table S7 (Supporting Information). This setup simplifies task complexity but demands greater data collection effort, making BioBridge's success particularly notable.

**Table 1:** Few‐shot performance comparison of meta‐unseen‐protein splitting on the BindingDB and BinSNAP datasets (**Best**, Second Best). Data are expressed as mean ± standard deviation.

Dataset	BindingDB(Meta Unseen Protein)			BioSNAP(Meta Unseen Protein)		
Metric	AUROC	AUPRC	ACC	AUROC	AUPRC	ACC
DrugBAN^[^ ^25^ ^]^	0.5573 ± 0.0137*	0.5723 ± 0.0181*	0.5281 ± 0.0060*	0.5779 ± 0.0175*	0.7219 ± 0.0203*	0.4876 ± 0.0125*
Setting	1‐shot					
MAML++^[^ ^26^ ^]^	0.6200 ± 0.0095	0.6060 ± 0.0053*	0.5894 ± 0.0119*	0.5884 ± 0.0136*	0.5659 ± 0.0132*	0.5594 ± 0.0086*
Protypes^[^ ^27^ ^]^	0.7079 ± 0.0213*	0.7080 ± 0.0239	0.6443 ± 0.0148	0.6536 ± 0.0114*	0.6455 ± 0.0136*	0.6080 ± 0.0071*
MetaOptNet^[^ ^28^ ^]^	0.7061 ± 0.0033*	0.6898 ± 0.0025*	0.6481 ± 0.0103*	0.6429 ± 0.0225	0.6254 ± 0.0225*	0.6053 ± 0.0161
ANIL^[^ ^29^ ^]^	0.7046 ± 0.0073	0.6994 ± 0.0082	0.6441 ± 0.0034*	0.6476 ± 0.0067*	0.6379 ± 0.0095*	0.6060 ± 0.0054*
BioBridge_*Meta*_	**0.7488 ± 0.0073**	**0.7545 ± 0.0075**	**0.6631 ± 0.0051**	**0.6755 ± 0.0073**	**0.6707 ± 0.0073**	**0.6237 ± 0.0040**
Setting	3‐shot					
MAML++^[^ ^26^ ^]^	0.7258 ± 0.0194	0.7146 ± 0.0208	0.6670 ± 0.0156*	0.685 ± 0.038*	0.6689 ± 0.0424*	0.6362 ± 0.0255*
Protypes^[^ ^27^ ^]^	0.7838 ± 0.0149*	0.7840 ± 0.0172*	0.7028 ± 0.0128*	0.7352 ± 0.0055*	0.7295 ± 0.0036*	0.6694 ± 0.0088*
MetaOptNet^[^ ^28^ ^]^	0.8099 ± 0.0136*	0.7903 ± 0.0160*	**0.7392 ± 0.0108** *	0.7603 ± 0.0087*	0.7422 ± 0.0058*	**0.6975 ± 0.0123** *
ANIL^[^ ^29^ ^]^	0.7704 ± 0.0182*	0.7682 ± 0.0098	0.6964 ± 0.0107*	0.7116 ± 0.0031*	0.695 ± 0.0016*	0.6612 ± 0.0024*
BioBridge_*Meta*_	**0.8227 ± 0.0023**	**0.8227 ± 0.0029**	0.7314 ± 0.0041	**0.7661 ± 0.0076**	**0.7664 ± 0.0084**	0.6910 ± 0.0057
Setting	5‐shot					
MAML++^[^ ^26^ ^]^	0.7452 ± 0.0196*	0.7392 ± 0.0243*	0.6801 ± 0.0135*	0.7251 ± 0.0341*	0.7055 ± 0.0353*	0.6682 ± 0.0244*
Protypes^[^ ^27^ ^]^	0.8200 ± 0.0104*	0.8205 ± 0.0114*	0.7353 ± 0.0085*	0.7718 ± 0.0058*	0.7674 ± 0.0073*	0.6983 ± 0.0031*
MetaOptNet^[^ ^28^ ^]^	0.8524 ± 0.0068	0.8381 ± 0.0082*	**0.7703 ± 0.0073**	0.7880 ± 0.0050*	0.7734 ± 0.0027*	0.7111 ± 0.0059*
ANIL^[^ ^29^ ^]^	0.7933 ± 0.0019*	0.7784 ± 0.0089*	0.7209 ± 0.0122*	0.7635 ± 0.013*	0.7560 ± 0.0143*	0.6934 ± 0.0121
BioBridge_*Meta*_	**0.8527 ± 0.0006**	**0.8555 ± 0.0007**	0.7624 ± 0.0016	**0.8030 ± 0.0006**	**0.8052 ± 0.0022**	**0.7235 ± 0.0045**

* Significantly different (p < 0.05) from the corresponding BioBridge metric value; one‐way analysis of variance (ANOVA).

In summary, the BioBridge pipeline builds upon generalized binding principles established during the inductive phase and leverages weakly associated annotations for associative learning, enabling accurate predictions of unknown drug‐target interactions. Additionally, BioBridge relies exclusively on sequence data, allowing efficient and rapid training and deployment tailored to specific scenarios (Figure S1, Supporting Information). These features underscore its potential for practical applications in drug discovery.

### 2.4 Interpretability Analysis

How does BioBridge benefit from both inductive and associative learning? Has BioBridge captured the fundamental chemical principles that determine binding? To address these questions, we visualized the learned representations of BioBridge and conducted interpretative analyses of its results on two crystal structures that were unseen during training.

**Figure 3:**
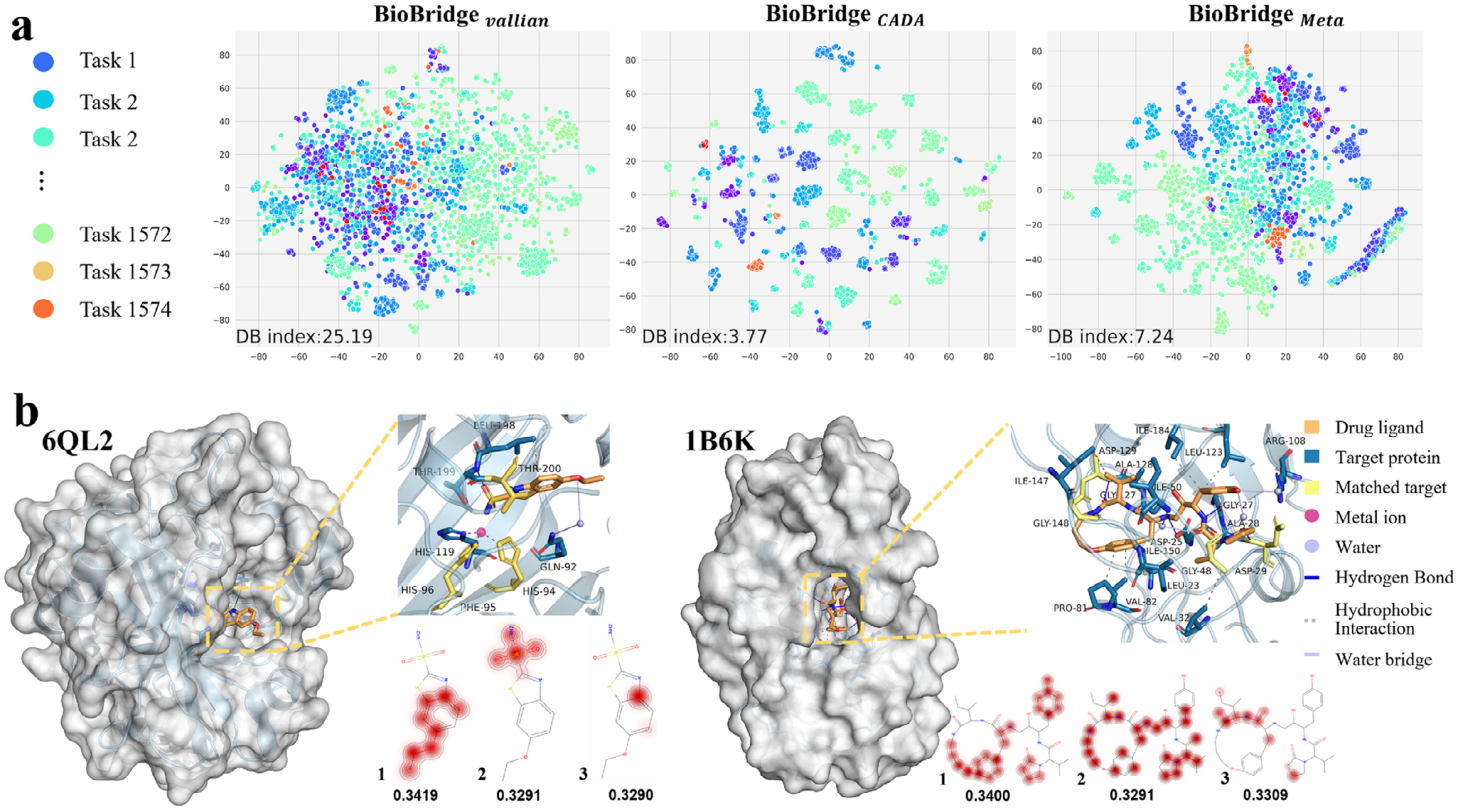
a) The t‐SNE visualization presents drug‐target pairs across various tasks, with lower Davies–Bouldin (DB) indices indicating superior performance. b) The visualization of drug ligand and target binding pocket attention is generated using Rdkit^[^ ^21^ ^]^ for drug molecule mapping and PyMOL^[^ ^42^ ^]^ for target plotting. PLIP^[^ ^43^ ^]^ is utilized to plot the forces between drug targets. The molecular diagram highlights the top 20% of model‐concerned positions in red, while the target docking diagram depicts the model's focus in yellow, aligning with actual interacting residues.

To evaluate the impact of cross‐domain pre‐training on knowledge transfer, we compare the performance of three vanilla BioBridge, BioBridge_*CADA*_, and BioBridge_*Meta*_ in a 5‐shot scenario, using the BindingDB dataset to extract drug‐target binding features for an unknown protein task. We apply the t‐SNE algorithm^[^ ^44^ ^]^ to reduce these features to two dimensions, as shown in **Figure** **3**a. The Davies–Bouldin index^[^ ^45^ ^]^ is used to assess the classification effectiveness across meta‐tasks. Our results show that vanilla BioBridge struggles to distinguish protein types based on drug‐binding properties, highlighting the challenges of generalizing to new tasks. In contrast, BioBridge_*CADA*_, which incorporates the CADA module, effectively identifies variations in binding modes, improving knowledge transfer to new protein targets. Although BioBridge_*Meta*_ exhibits a slight decrease in protein discrimination after meta‐training, it still outperforms vanilla BioBridge, likely due to its focus on associative ability during meta‐training.

BioBridge utilizes attention mechanisms to encode drug‐target interactions, allowing the model to assess the impact of substructures on binding. We examined high‐resolution X‐ray structures of human protein targets from PDB entries 6QL2^[^ ^46^ ^]^ and 1B6K,^[^ ^47^ ^]^ selecting structures with co‐crystallized ligands (pIC50 ⩽ 100 nM) that were not part of the training set. Figure 3b shows the ligand‐protein interaction maps, with the top 20% of atoms, based on bilinear attention coefficients, highlighted in red across the model's three hierarchical domains. Residues of the target protein that align with the X‐ray structures are marked in yellow.

For 6QL2, the first domain focuses on the benzene ring and ethoxy group, which engage in hydrophobic interactions^[^ ^48^ ^]^ and potential water‐mediated bridging.^[^ ^49^ ^]^ The second domain highlights the sulfonamide group, which is known to participate in hydrogen bonding through both the sulfonyl oxygens and the nitrogen atom, contributing to polar interactions within the binding pocket.^[^ ^50^ ^]^ The third domain targets the benzothiazole bridgehead carbon atom, revealing no specific interactions with the protein. For 1B6K, BioBridge's first domain identifies the N‐heterocyclic bi‐ring of PI5, with hydrophobic interactions and hydrogen bonding.^[^ ^51^, ^52^ ^]^ The second domain detects secondary amines, which serve as a hydrogen bond donor and may also participate in hydrophobic or π‐related interactions.^[^ ^53^ ^]^ The third domain involves the nitrogen atom as a hydrogen bond donor, with oxygen acceptors and water‐mediated bridging interactions.^[^ ^49^ ^]^ Despite the challenges in interpreting protein sequences, BioBridge successfully identifies key residues involved in binding for both 6QL2 and 1B6K, demonstrating its potential for capturing interaction principles for unknown drug‐target pairs.

### 2.5 Virtual Screening with BioBridge

Having established the effectiveness and interpretability of BioBridge, we evaluate its practical application in virtual screening across two protein target families: the epidermal growth factor receptor family (EGFR: HER1, HER2, HER3), crucial for cellular processes, and the adenosine receptor family (AR: AA1R, AA2AR, AA2BR), essential for cellular signaling and immune responses. This evaluation demonstrates BioBridge's high extensibility in real‐world scenarios, requiring chemists to reference only a few homologous protein‐binding interactions to predict novel drug‐target binding pairs.

**Figure 4:**
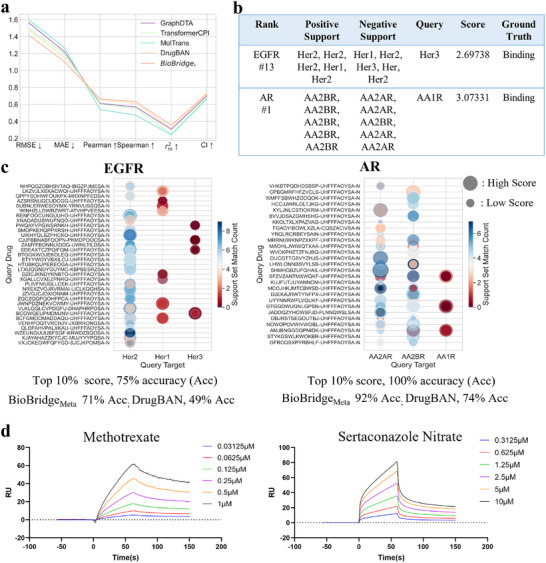
a) Mean performance on the PDBBind v2020 datasets is compared, with ↑ indicating higher scores are favorable and ↓ signifying the opposite. b) Virtual screening examples from tyrosinase and adenosine receptor families feature top 10% scoring compounds with a limited query protein in the support set. c) In these virtual screenings, color represents the frequency of the query protein in the support set, and bubble size corresponds to the magnitude of the score. Top 10% meta‐tasks for adenosine receptors even achieved 100% accuracy. BioBridge surpassed DrugBAN in traditional classification tasks, demonstrating superior adaptability to novel drug‐target pairs. d) Surface plasmon resonance sensorgrams showing the binding of nitraconazole nitrate and methotrexate to AA1R. Both compounds exhibit dose‐dependent binding profiles consistent with a 1:1 Langmuir model, with *K*_*D*_ values of 2.23 × 10^−6^ M and 5.48 × 10^−7^ M, respectively.

To enhance screening accuracy, we develop BioBridge_*r*_, trained on PDB2020 data, integrating seamlessly with BioBridge via a single‐layer decoding process. We also compare BioBridge_*r*_ with several state‐of‐the‐art models in PDB2020, as depicted in **Figure** **4**a. BioBridge_*r*_ demonstrates comprehensive superiority, affirming the robustness and broad applicability of the BioBridge encoder. A calculated score determines the drug's binding effectiveness, the product of BioBridge's binding likelihood, and BioBridge_*r*_'s binding strength, squared to emphasize the initial prediction: $y_{c}^{2}\times y_{r}$. Notably, the assessed proteins are excluded from our training data.

We randomly sampled 200 tasks from the epidermal growth factor and adenosine receptor families. InChI Keys represent drugs, and all of them are reported and validated entities. We calculate the predicted scores for each task using the methods described above and tally the occurrence of query proteins in the support set for each task, as shown in Figure 4b. For tasks with adenosine receptor family prediction scores in the top 10%, accuracies achieve 100%. Notably, Figure 4c shows that the binding status of queried drug‐target pairs can be accurately predicted with minimal examples, highlighting the practical utility of BioBridge in drug development with limited annotation data. Compared to conventional inductive DTI approaches, we report the classification accuracies of BioBridge_*Meta*_ across all tasks alongside the state‐of‐the‐art method DrugBAN. BioBridge_*Meta*_ achieves approximately 20% higher accuracy than DrugBAN on previously unseen targets, unequivocally demonstrating its pipeline's potential in addressing novel drug‐target pairs.

To systematically evaluate the generalization ability of BioBridge, we screened a curated subset of FDA‐approved small molecules from the ZINC database against the AA1R receptor using our trained model. We selected the top two predicted binders from this screenNitraconazole nitrate (CAS: 99592‐32‐2) and Methotrexate (CAS: 59‐05‐2) for experimental validation. Binding affinities were measured using surface plasmon resonance (SPR) on a Biacore 1K system with CM5 sensor chips. Recombinant AA1R was immobilized using standard EDC/NHS coupling in 10 mM sodium acetate buffer (pH 5.0) at a flow rate of 10 µL/min for 420 s. A reference flow cell was prepared in parallel without protein immobilization. Compounds were serially diluted in PBS with 5% DMSO and injected at 10 µL/min for 150 s, followed by regeneration with 10 mM glycine‐HCl (pH 2.0) for 5 minutes. Sensorgrams were double‐referenced and fitted to a 1:1 Langmuir binding model using Biacore Insight software (v2.0, Cytiva). For more details, please refer to Section S12 (Supporting Information).

The dissociation constants ($K_{D}$) were determined to be 2.23 × 10^−6^ M for Nitraconazole nitrate and 5.48 × 10^−7^ M for Methotrexate (Figure 4d), indicating moderate to high binding affinities that support our model's predictions. These biophysical measurements confirm BioBridge's ability to identify previously unreported drug‐target interactions under a cold‐start setting. Notably, neither interaction was documented in PubChem BioActivity entries (accessed May 10, 2025), underscoring the model's potential for discovering novel and high‐confidence drug candidates when empirical data are sparse.

### 2.6 Ablation Study

To demonstrate the added value of individual modules and strategies in BioBridge, we also conduct extensive ablation studies to evaluate their effectiveness.

As shown in Table S9 (Supporting Information), an ablation study assesses the Multi‐scale Awareness, Stem, and GAU modules of the BioBridge multi‐level encoder. Removing these components reveals their impact, with BioBridge maintaining superior performance across all metrics. Multi‐level feature models outperformed single‐level ones, highlighting their significance in knowledge transfer. As detailed in Supplementary Figure S3, the CADA module outperforms CDAN in cross‐domain tasks, with BioBridge_*CADA*_ achieving the best results, followed by DrugBAN_*CADA*_ and BioBridge_*CDAN*_. This hierarchy highlights that category‐aware alignment distinguishing between positive and negative interactions during adaptation boosts performance more effectively than generic domain adaptation, such as CDAN. Notably, even with the same encoder, CADA better preserves discriminative features, confirming that task‐tailored cross‐domain strategies are critical for capturing drug‐target interactions, outweighing the impact of the underlying model architecture.

We also explore several key modules within the setting of associative tasks, including Multi‐level Awareness (MA), Inductive Training (LOAD), Dynamic Prototype (DGL), and Focal Loss (FL). Removing the MA module reverts it to a feature merge operation. As shown in **Tables** **2**, the performance of the models on various few‐shot tasks across the BioSNAP and BindingDB datasets decreases after removing different modules, validating the effectiveness of our design. The removal of Focal Loss also has a significant impact on performance, indicating that an adaptive focus on challenging samples is beneficial in drug discovery where the learning difficulty varies.

**Table 2:** Ablation experiment of BioBridge encoder on meta unseen protein splitting of BindingDB and BioSNAP dataset(**Best**, Second Best). Data are expressed as mean ± standard deviation.

Dataset				BindingDB (Meta unseen protein)								
Setting				1‐ shot			3‐ shot			5‐ shot		
MA	DGL	FL	Load	AUROC	AUPRC	ACC	AUROC	AUPRC	ACC	AUROC	AUPRC	ACC
✓	✓	✓	✓	**0.7488 ± 0.0073**	**0.7545 ± 0.0075**	0.6631 ± 0.0051	**0.8227 ± 0.0023**	**0.8227 ± 0.0029**	**0.7314 ± 0.0041**	**0.8527 ± 0.0006**	**0.8555 ± 0.0007**	**0.7624 ± 0.0016**
✓	✗	✓	✓	0.7412 ± 0.0101	0.7467 ± 0.0104	0.6659 ± 0.0075	0.8205 ± 0.0034	0.8245 ± 0.0052	0.7303 ± 0.0065	0.8465 ± 0.0031	0.8489 ± 0.0028	0.7535 ± 0.0042
✓	✓	✗	✓	0.7194 ± 0.0182	0.7212 ± 0.0210	0.6541 ± 0.0098	0.7966 ± 0.0031	0.7991 ± 0.0003	0.7132 ± 0.0052	0.8306 ± 0.0130	0.8322 ± 0.0104	0.7458 ± 0.0099
✓	✗	✗	✓	0.7079 ± 0.0213	0.7080 ± 0.0239	0.6443 ± 0.0148	0.7838 ± 0.0149	0.7840 ± 0.0172	0.7028 ± 0.0128	0.8200 ± 0.0104	0.8205 ± 0.0114	0.7353 ± 0.0085
✓	✓	✓	✗	0.7232 ± 0.0042	0.7258 ± 0.0054	0.6531 ± 0.0036	0.7924 ± 0.0043	0.7945 ± 0.0040	0.7091 ± 0.0049	0.8351 ± 0.0085	0.8351 ± 0.0071	0.7483 ± 0.0106
✓	✓	✗	✗	0.6777 ± 0.0083	0.6743 ± 0.0091	0.6224 ± 0.0076	0.7647 ± 0.0234	0.7652 ± 0.0225	0.6880 ± 0.0183	0.7822 ± 0.1417	0.7808 ± 0.0176	0.7055 ± 0.0095
✓	✗	✓	✗	0.7279 ± 0.0109	0.7304 ± 0.0105	0.6557 ± 0.0091	0.7880 ± 0.0107	0.7899 ± 0.0109	0.7063 ± 0.0081	0.8259 ± 0.0048	0.8259 ± 0.0042	0.7407 ± 0.0067
✓	✗	✗	✗	0.6983 ± 0.0074	0.6996 ± 0.0085	0.6354 ± 0.0065	0.7498 ± 0.0190	0.7492 ± 0.0175	0.6783 ± 0.0157	0.7760 ± 0.0165	0.7726 ± 0.0201	0.7022 ± 0.0093
✗	✓	✓	✓	0.7388 ± 0.0062	0.7463 ± 0.0103	0.6629 ± 0.0019	0.8140 ± 0.0011	0.8135 ± 0.0055	0.7292 ± 0.0010	0.8433 ± 0.0002	0.8448 ± 0.0002	0.7547 ± 0.0002
✗	✗	✓	✓	0.7398 ± 0.0090	0.7385 ± 0.0108	**0.6701 ± 0.0048**	0.8043 ± 0.0066	0.8028 ± 0.0076	0.7222 ± 0.0056	0.8349 ± 0.0042	0.8362 ± 0.0030	0.7466 ± 0.0043
✗	✓	✗	✓	0.7063 ± 0.0256	0.7027 ± 0.0284	0.6475 ± 0.0162	0.7979 ± 0.0023	0.7974 ± 0.0004	0.7162 ± 0.0051	0.8205 ± 0.0075	0.8204 ± 0.0072	0.7358 ± 0.0067
✗	✗	✗	✓	0.7355 ± 0.0049	0.7362 ± 0.0065	0.6641 ± 0.0032	0.7878 ± 0.0008	0.7833 ± 0.0001	0.7108 ± 0.0012	0.8033 ± 0.0061	0.8002 ± 0.0038	0.7257 ± 0.0066
✗	✓	✓	✗	0.7191 ± 0.0172	0.7201 ± 0.0193	0.6508 ± 0.0118	0.7988 ± 0.0012	0.8003 ± 0.0030	0.7154 ± 0.0007	0.8272 ± 0.0039	0.8266 ± 0.0042	0.7413 ± 0.0018
✗	✓	✗	✗	0.6660 ± 0.0062	0.6590 ± 0.0067	0.6151 ± 0.0044	0.7362 ± 0.0068	0.7296 ± 0.0074	0.6702 ± 0.0039	0.7718 ± 0.0088	0.7671 ± 0.0096	0.6971 ± 0.0073
✗	✗	✓	✗	0.7119 ± 0.0087	0.7115 ± 0.0098	0.6451 ± 0.0074	0.7867 ± 0.0048	0.7865 ± 0.0042	0.7049 ± 0.0060	0.8194 ± 0.0027	0.8193 ± 0.0035	0.7344 ± 0.0023
✗	✗	✗	✗	0.6772 ± 0.0029	0.6704 ± 0.0044	0.6239 ± 0.0021	0.7053 ± 0.0023	0.6993 ± 0.0007	0.6465 ± 0.0043	0.7505 ± 0.0028	0.7447 ± 0.0006	0.6828 ± 0.0020

Dataset				BioSNAP (Meta unseen protein)								
Setting				1‐ shot			3‐ shot			5‐shot		
MA	DGL	FL	Load	AUROC	AUPRC	ACC	AUROC	AUPRC	ACC	AUROC	AUPRC	ACC
✓	✓	✓	✓	**0.6755 ± 0.0073**	0.6707 ± 0.0073	0.6237 ± 0.0040	**0.7661 ± 0.0076**	**0.7664 ± 0.0084**	**0.6910 ± 0.0057**	**0.8030 ± 0.0006**	**0.8052 ± 0.0022**	**0.7215 ± 0.0045**
✓	✗	✓	✓	0.6755 ± 0.0076	**0.6710 ± 0.0104**	**0.6238 ± 0.0065**	0.7630 ± 0.0071	0.7623 ± 0.0072	0.6889 ± 0.0065	0.7948 ± 0.0051	0.7937 ± 0.0070	0.7150 ± 0.0065
✓	✓	✗	✓	0.6542 ± 0.0111	0.6456 ± 0.0133	0.6042 ± 0.0057	0.7435 ± 0.0046	0.7397 ± 0.0019	0.6779 ± 0.0048	0.7781 ± 0.0060	0.7746 ± 0.0069	0.7045 ± 0.0015
✓	✗	✗	✓	0.6536 ± 0.0114	0.6455 ± 0.0136	0.6080 ± 0.0071	0.7352 ± 0.0055	0.7295 ± 0.0036	0.6694 ± 0.0088	0.7718 ± 0.0058	0.7674 ± 0.0073	0.6983 ± 0.0031
✓	✓	✓	✗	0.6731 ± 0.0097	0.6675 ± 0.0110	0.6252 ± 0.0043	0.7434 ± 0.0165	0.7400 ± 0.0178	0.6753 ± 0.0113	0.7766 ± 0.005	0.7744 ± 0.0046	0.7028 ± 0.0068
✓	✓	✗	✗	0.6532 ± 0.0236	0.6468 ± 0.0243	0.6072 ± 0.0173	0.7024 ± 0.0162	0.6977 ± 0.0175	0.6463 ± 0.0123	0.7246 ± 0.0409	0.7206 ± 0.0428	0.6620 ± 0.0296
✓	✗	✓	✗	0.6726 ± 0.0032	0.6690 ± 0.0018	0.6170 ± 0.0037	0.7390 ± 0.0206	0.7363 ± 0.0210	0.6716 ± 0.0112	0.7759 ± 0.0062	0.7719 ± 0.0062	0.7001 ± 0.0093
✓	✗	✗	✗	0.6496 ± 0.0165	0.6432 ± 0.0152	0.6026 ± 0.0116	0.7029 ± 0.0319	0.6986 ± 0.0311	0.6444 ± 0.0224	0.7329 ± 0.0242	0.7309 ± 0.0259	0.6649 ± 0.0197
✗	✓	✓	✓	0.6762 ± 0.0133	0.6691 ± 0.0156	0.6264 ± 0.0078	0.7585 ± 0.0050	0.7489 ± 0.0035	0.6933 ± 0.0038	0.7898 ± 0.0015	0.7803 ± 0.0006	0.7202 ± 0.0006
✗	✗	✓	✓	0.6730 ± 0.0219	0.6645 ± 0.0253	0.6225 ± 0.0160	0.7492 ± 0.0045	0.7411 ± 0.0065	0.6838 ± 0.0072	0.7842 ± 0.0033	0.7756 ± 0.0027	0.7151 ± 0.0025
✗	✓	✗	✓	0.6717 ± 0.0189	0.6608 ± 0.0205	0.6236 ± 0.0152	0.7386 ± 0.0052	0.7286 ± 0.0058	0.6769 ± 0.0044	0.7711 ± 0.004	0.7615 ± 0.0074	0.7078 ± 0.0026
✗	✗	✗	✓	0.6772 ± 0.0175	0.6679 ± 0.0202	0.6249 ± 0.0121	0.7350 ± 0.0063	0.7241 ± 0.0066	0.6738 ± 0.0057	0.7613 ± 0.004	0.7498 ± 0.0022	0.6958 ± 0.0022
✗	✓	✓	✗	0.6529 ± 0.0079	0.6454 ± 0.0072	0.6090 ± 0.0098	0.7353 ± 0.0050	0.7284 ± 0.0062	0.6689 ± 0.0037	0.7475 ± 0.0067	0.7391 ± 0.0062	0.6861 ± 0.0017
✗	✓	✗	✗	0.6335 ± 0.0258	0.6280 ± 0.0226	0.5939 ± 0.0276	0.6835 ± 0.0333	0.6752 ± 0.0276	0.6296 ± 0.0264	0.7134 ± 0.0201	0.7065 ± 0.0193	0.6517 ± 0.0156
✗	✗	✓	✗	0.6459 ± 0.0078	0.6395 ± 0.0091	0.6041 ± 0.0030	0.7260 ± 0.0227	0.7185 ± 0.0258	0.6628 ± 0.0163	0.7511 ± 0.0108	0.7425 ± 0.0126	0.6859 ± 0.0036
✗	✗	✗	✗	0.6270 ± 0.0217	0.6188 ± 0.0221	0.5872 ± 0.0175	0.6709 ± 0.0372	0.6665 ± 0.0369	0.6184 ± 0.0299	0.7184 ± 0.0080	0.7130 ± 0.0081	0.6570 ± 0.0044

## 3 Conclusion

Understanding protein‐drug interactions for proteins and drugs not present in training data is a critical challenge in drug discovery. To address this challenge, we present BioBridge, a workflow that emulates chemists' induction‐association approach by leveraging transferable interaction patterns and limited binding annotations to predict novel drug‐target interactions. BioBridge overcomes the limitations of prior methods, which struggle to balance these elements, and improves the understanding of protein variability. The framework employs three core strategies: (1) a multi‐level awareness mechanism capturing hierarchical interaction patterns, (2) category‐aware adversarial learning to distill transferable principles, and (3) dynamic prototype learning to resolve annotation inconsistencies.

As a proof‐of‐concept, BioBridge demonstrates a 30% improvement in AUROC over traditional inductive methods under sparse annotation scenarios. Its interpretable multi‐level binding analysis enables cost‐effective pharmacophore profiling and binding site insights. Crucially, BioBridge generalizes to unseen drug‐target pairs using only sequence data and minimal annotations, highlighting its scalability for drug discovery. Three variants are proposed: BioBridge_*vanilla*_ for well‐characterized pairs, BioBridge_*CADA*_ for novel targets, and BioBridge_*Meta*_ for scenarios with scarce target‐binding annotation resources.

However, BioBridge has some limitations. Its multi‐level encoder is slower due to hierarchical interaction extraction, and its modeling of intramolecular forces is simplistic. Additionally, it does not yet incorporate 3D protein structure data, which is limited for most proteins. Finally, BioBridge's validation on BindingDB, BioSNAP, and Human datasets highlights its potential, yet broader applications require testing on complex scenarios involving polypharmacology or multi‐target interactions.

BioBridge's modular prediction pipeline, grounded in inductive and associative learning frameworks, exhibits inherent theoretical extensibility to accommodate multifaceted enhancements. Future research could refine its capacity to distill interaction patterns through advanced inductive learning strategies or context‐aware associative reasoning while leveraging cost‐effective 3D structural insights derived from AlphaFold‐predicted geometries. Systematic pre‐training on large‐scale, heterogeneous datasets (e.g., ChEMBL, DrugBank) would further strengthen its robustness against polypharmacological and multi‐target binding scenarios. Additionally, integrating neural architecture search via meta‐learning could overcome the limitations of manually designed models by optimizing architecture, feature learning, and prototype reasoning in a unified framework. These synergistic extensions position BioBridge as an adaptable pipeline for bridging scalable theoretical principles with practical generalizability in drug discovery.

## 4 Experimental Section

The core innovation of BioBridge lies in its inductive‐associative framework, which mirrors a scientist's approach of combining accumulated knowledge with limited reference to make novel predictions about drug‐target interactions.

During the inductive phase, BioBridge adopts a multi‐level perception strategy inspired by human reasoning. This phase further employs adversarial learning to uncover interaction principles that are transferable to previously unseen scenarios.

In the associative phase, BioBridge replicates the process of consulting related references. It uses unsupervised clustering to identify weakly related interaction annotations and enhances the model's reasoning capabilities through dynamic prototype‐based meta‐learning.

### BioBridge Multi‐Levels Encoder

The BioBridge encoder (Figure S4a, Supporting Information) facilitates the analysis of protein‐molecule interactions by leveraging protein sequences and molecular graphs to capture diverse interaction patterns. Inspired by human multi‐level understanding and the robust transferability of shallow features in cross‐domain tasks, BioBridge adopts a multi‐level perceptive encoder.^[^ ^22^, ^23^ ^]^ This design concludes interaction information incrementally, enabling broader inference of binding patterns and improved cross‐domain adaptability.^[^ ^54^ ^]^ More detailed architectural specifics are provided in the Section S14 (Supporting Information).

The BioBridge encoder consists of a protein encoder $f^{p}$ and a molecule encoder, $f^{p}$ each divided into l stages. At stage, l the protein and molecule representations, $P_{i}$ and $D_{i}$, respectively, are updated using transformation functions $f_{i}^{p}$ and $f_{i}^{d}$:

(1) $$\begin{array}{c} P_{i}=f_{i}^{p}\left(P_{i-1}\right),\;D_{i}=f_{i}^{d}\left(D_{i-1}\right) \end{array}$$

This stage‐wise process allows the encoder to progressively capture granular interaction patterns between proteins and molecules, reflecting their intrinsic properties.

To model protein‐molecule interactions, a binding interaction encoder $f_{b}$ fuses the representations $P_{i}$ and $D_{i}$ at each stage i, producing interaction features $I_{i}$:

(2) $$I_{i}=f_{i}^{b}\left(P_{i},D_{i}\right)$$

These features encapsulate both local and cross‐level interaction patterns, offering a comprehensive representation of drug‐target pairs.

To identify the most relevant interaction patterns for drug‐target interaction prediction, a selection en $f_{s}$ integrates features across all stages. The final interaction pattern O is computed as:

(3) $$O=f^{s}\left(\sum_{i=1}^{I}I_{i}\right)$$

This process aggregates and refines the interaction features, enhancing adaptability and generalization to complex drug discovery scenarios.

### Inductive‐Learning Stage

The inductive learning phase focuses on the model's ability to generalize and transfer interaction patterns, which is assessed by its zero‐shot accuracy in predicting novel drug‐target interactions. Drug–target pairs are denoted as $X=\{x_{1},x_{2},\text{…}\;,x_{n}\}$, where each pair $x_{i}=\left\{D_{i},P_{i}\right\}$ consists of a drug $D_{i}$ and its target protein $P_{i}$. The corresponding interaction labels are $Y=\{y_{1},y_{2},\text{…}\;,y_{n}\}$. The objective is to develop a model f that predicts the interaction y for a novel drug‐target pair x, such that f(x)=y.

The multi‐level perceptron encoder mimics the comprehensive understanding scientists have of the criteria for drug‐target binding. However, applying these criteria to new drug development poses challenges due to significant differences across proteins. To overcome these challenges, we propose a Category‐Aware Adversarial Training (CADA) strategy during the inductive phase to identify interaction criteria applicable to novel drug‐target pairs. This approach involves transferring interaction knowledge from a source domain $\text{Source}={\left\{\left(x_{i},y_{i}\right)\right\}}_{i=1}^{N_{s}}$, which includes $N_{s}$ labeled drug‐protein pairs, to a target domain $\text{Target}={\left\{\left(x_{i}\right)\right\}}_{i=1}^{N_{t}}$, consisting of $N_{t}$ unlabeled pairs.

CADA distinguishes the domain origin of interaction representations while aligning positive and negative instances from both domains within a shared feature space, thereby preserving and transferring key interaction criteria. While CADA utilizes the adversarial alignment approach of CDAN,^[^ ^25^ ^]^ it overcomes CDAN's limitationwhere a single discriminator aligns the overall feature distribution, potentially blending positive and negative patterns in the target domain. Drawing inspiration from pharmacologists' practice of analyzing active and inactive compounds separately, CADA implements a dual‐channel discriminator to process positive and negative interaction samples independently.

Figure S4d (Supporting Information) depicts the interaction features $O_{s}$ and $O_{t}$ extracted by multi‐levels aware encoder, along with predicted probabilities $\hat{y}=\left\{P_{s}^{1},P_{s}^{2},P_{t}^{1},P_{t}^{2}\right\}$. A Gradient Reversal Layer (GRL) is applied to produce reversed gradients $\{R_{s},R_{t}\}$, which are scaled by the predicted probabilities and input into the discriminator $D=\{D_{0},D_{1}\}$, where 0 indicates negative instances, and 1 indicates positive:

(4) $$\begin{array}{c} R_{s}=GRL\left(O_{s}\right),\;R_{t}=GRL\left(O_{t}\right) \end{array}$$

(5) $$\begin{array}{c} L_{d}\left(\theta_{d}\right)=\frac{1}{n}\sum_{k=1}^{2}\sum_{x_{i}\in R_{s}\cup R_{t}}d_{i}\log\left(D_{k}\left({\hat{y}}_{i}^{k}x_{i}\right)\right) \end{array}$$

Here, $D_{k}$ differentiates between domains, and $d_{i}$ represents the domain label. The model's backbone parameters $\hat{\theta_{f}}$ are optimized to minimize the cross‐entropy loss $L_{s}$ on the source domain, while the discriminator's parameters $\hat{\theta_{d}}$ aim to maximize the adversarial loss $L_{d}$. These competing objectives are balanced by a hyperparameter λ:

(6) $$\begin{array}{cc} L(\theta_{f},\theta_{d}) & =L_{s}\left(\theta_{f}\right)+\lambda L_{d}\left(\theta_{d}\right)\\ \hat{\theta_{f}} & =\min L_{s}\left(\theta_{f}\right),\;\hat{\theta_{d}}=\max L_{d}\left(\theta_{d}\right) \end{array}$$

### Association‐Learning Stage

We ensure that the meta‐learning encoder retains transferable interaction principles learned during the inductive phase through parameter passing, enabling the expansion of the model's associative capabilities.

In the association stage, the model's ability to infer novel drug‐target interactions is evaluated based on the accuracy of few‐shot predictions. Unlike traditional methods that rely on high‐quality reference data, our approach addresses real‐world challenges by focusing on sparsely annotated or unknown drug‐target pairs. A formal definition of meta‐learning is provided in Section S5 (Supporting Information).

To simulate realistic drug discovery scenarios, we extend the source and target domains by creating distinct cross‐domain meta‐tasks. These tasks are constructed using unsupervised clustering of broader protein categories rather than individual protein targets,^[^ ^18^ ^]^ ensuring significant divergence between training and test data. This approach reflects how researchers derive insights from weakly related references when exploring new drug‐target interactions. Further details of the meta‐task construction are included in Section S6 (Supporting Information).

The association stage trains a meta‐learning model $f_{\mathrm{meta}}$ to predict the interaction *y* for a query drug‐target pair Q, given a set of weakly related reference pairs $S=\{x_{1},x_{2},\text{…}\;,x_{n}\}$. Each task comprises support sets $S=\{(X_{s},Y_{s})\}$ and query sets $Q=\{(X_{q},Y_{q})\}$. BioBridge adopts a metric‐based strategy for task adaptation via dynamic prototype learning, avoiding gradient‐based methods like MAML that require costly inner‐loop optimization and are sensitive to learning rates. This sensitivity limits their stability and scalability in low‐resource biological settings. Instead, BioBridge aggregates interaction features into category prototypes using affine attention and matches query samples based on cosine similarity, facilitating generalization across diverse proteins.

As illustrated in Figure S4e (Supporting Information), the model is trained on N distinct seen tasks

$T=\{T_{1}^{\text{seen}},\text{…},T_{N}^{\text{seen}}\}$, organized by protein or molecule clusters. Each support set contains k positive and negative samples (2‐way k‐shot). The meta‐interaction encoder $f_{\mathrm{meta}}$ derives feature representations for support interactions $O_{s}\in R^{N\times 2k\times d}$ and query interactions $O_{q}\in R^{N\times k_{q}\times d}$. These features are concatenated through tensor expansion operations:

(7) $$\begin{array}{cc} O_{s}^{\prime } & =\text{Expand}\left(O_{s},\text{dim}=1\right)\in R^{N\times k_{q}\times 2k\times d}\\ O_{c} & =\left[O_{q}⊗1_{2k};O_{s}^{\prime }\right]\in R^{N\times k_{q}\times \left(2k+1\right)\times d} \end{array}$$

where Expand(·) denotes dimension expansion with replication, [·;·] represents concatenation along the third dimension, and ⊗ indicates tensor broadcasting. The class prototypes P are computed through attention mechanisms:

(8) $$\begin{array}{cc} Q & =\sigma \left(W_{I}O_{c}\right)\circ \gamma_{1}+\beta_{1}\\ K & =\sigma \left(W_{I}O_{c}\right)\circ \gamma_{2}+\beta_{2}\\ A & ={\left[\text{ReLU}(QK^{⊤})\right]}^{⊙2} \end{array}$$

(9) $$\begin{array}{c} P\left[n,q,c\right]=\frac{1}{|O_{c}|}\sum_{i\in O_{c}}O_{s}^{\prime }\left[n,q,i,:\right]\cdot \frac{\exp(A[n,q,i])}{\sum_{j=1}^{2k}\exp\left(A\left[n,q,j\right]\right)} \end{array}$$

where σ denotes the SiLU activation function, ∘ represents element‐wise multiplication, and ⊙2 indicates element‐wise square.

To classify, $X_{q}$ we compute the cosine similarity between P and $X_{q}$ Recognizing that negative samples are easier to classify in drug‐target interactions, we use Focal Loss to emphasize harder‐to‐classify cases. This dynamically reduces the weight of easily distinguishable samples, focusing on challenging ones:

(10) $$\begin{array}{cc} s_{nqc} & =\cos(P\left[n,q,c\right],X_{q}\left[n,q\right])\\ p_{nq} & =\sum_{c\in \{0,1\}}\frac{\exp(s_{nqc})}{\sum_{c^{\prime }}\exp\left(s_{nqc^{\prime }}\right)}\cdot I\left(Y_{q}=c\right)\\ L_{f} & =-\alpha \sum_{n,q}{\left(1-p_{nq}\right)}^{\gamma }\log\left(p_{nq}\right) \end{array}$$

Here, I(·) is the Kronecker delta function, which equals 1 if $Y_{q}=c$ and 0 otherwise. The class weight α balances losses between positive and negative samples, while the modulation factor ${(1-p_{\mathrm{nq}})}^{\gamma }$ minimizes the impact of easily classified samples and emphasizes challenging ones. The final parameters are obtained by ${\hat{\theta }}_{m}=\arg\min_{\theta_{m}}L_{f}$.

### Statistical Analysis

All statistical analyses were performed using Python (version 3.8) with the SciPy and NumPy libraries. Results are reported as mean ± standard deviation unless otherwise specified. A one‐way analysis of variance (ANOVA) was used for group comparisons. A two‐sided hypothesis testing framework was applied with a significance threshold of P < 0.05. Statistical tests were chosen based on assumptions of normality and homoscedasticity. Sample sizes (n) varied depending on the dataset and experimental configuration; please refer to Supporting Information for details.

## Conflict of Interest

The authors declare no conflict of interest.

## Supporting information

Supporting Information

## Data Availability

The data that support the findings of this study are available from the corresponding author upon reasonable request.
