# Supplementary material for: Inductive‐Associative Meta‐learning Pipeline with Human Cognitive Patterns for Unseen Drug‐Target Interaction Prediction
Source: Adv Sci (Weinh). 2025 Jul 1;12(35):e06404. doi: 10.1002/advs.202506404 (PMC12462924; doi:10.1002/advs.202506404)
Supplement: Supplementary file 1 — Supporting Information [file ADVS-12-e06404-s001.pdf]

ADVANCED SCIENCE
Open Access

Supporting Information

for *Adv. Sci.*, DOI 10.1002/advs.202506404

Inductive-Associative Meta-learning Pipeline with Human Cognitive Patterns for Unseen Drug-Target Interaction Prediction

Xiaoqing Lian, Tianxu Lv, Jie Zhu, Shiyun Nie, Hang Fan, Guosheng Wu, Yunjun Ge, Hong Xu, Xiaoting Wang*, Lihua Li*, Xiangxiang Zeng* and Xiang Pan**

Supplementary Material for Inductive-Associative Meta-learning Pipeline with Human Cognitive Patterns for Unseen Drug-Target Interaction Prediction

Xiaoqing Lian Tianxu Lv Jie Zhu Shiyun Nie Hang Fan Guosheng Wu Yunjun Ge Hong Xu Xiaoting Wang* Lihua Li* Xiangxiang Zeng* Xiang Pan**

Xiaoqing Lian, Tianxu Lv, Jie Zhu, Shiyun Nie, Hang Fan, Xiang Pan
School of Artificial Intelligence and Computer Science, Jiangnan University, Wuxi 214122, Jiangsu, China
Email Address: xiangpan@jiangnan.edu.cn
Guosheng Wu, Yunjun Ge
Department of Basic Medical Science, Wuxi School of Medicine, Jiangnan University, Wuxi 214122, Jiangsu, China
Hong Xu
Department of Oncology, The Changshu Affiliated Hospital of Soochow University, Suzhou, Jiangsu, China
Email Address: 20214133016@stu.suda.edu.cn
Xiaoting Wang
Jiangnan University Medical Center (Wuxi No. 2 People's Hospital), Wuxi 214000, China.
Email Address: xiaotingwang@msn.com
Lihua Li
Institute of Biomedical Engineering and Instrumentation, Hangzhou Dianzi University, Hangzhou 310018, Zhejiang, China
Email Address: lilh@hdu.edu.cn
Xiangxiang Zeng
College of Information Science and Engineering, Hunan University, Changsha 410082, Hunan, China
Email Address: xzeng@hun.edu.cn
Xiang Pan
The PRC Ministry of Education Engineering Research Center of Intelligent Technology for Healthcare, Wuxi 214122, Jiangsu, China
Email Address: xiangpan@jiangnan.edu.cn

S1 Datasets

We utilize three key datasets for Drug-Target Interaction (DTI) research: BindingDB, BioSNAP, and Human. BindingDB is an online database that provides experimentally confirmed binding affinities between small molecules and proteins. It is known for its reliability. We employ a low-bias version developed by Bai [3]. The BioSNAP dataset, compiled from DrugBank by Huang [10] and Marinka [27], includes data on nearly 5,000 drugs and over 2,000 proteins. It is characterized by a balanced distribution of positive instances (known interactions) and randomly selected negative instances to ensure dataset equilibrium. The Human dataset, assembled by Liu [16], is noted for its high-quality negative samples derived from advanced computational screening methods. We use a balanced version of this dataset, containing an equal number of positive and negative samples. Additional details about these datasets are provided in Table S1.

Table S1: Experimental dataset statistics.

Dataset	Drugs	Targets	Interactions
BingdingDB [3]	14643	2623	49199
BioSNAP [9]	4510	2181	27464
Human [16]	2726	2001	6728

S2 Notations and descriptions

Table S2 represents variations and explanations of BioBridge.

Table S2: Variations and explanations of BioBridge.

Model Name	Explanation
BioBridge$_{vanilla}$	The foundational model employing multi-level encoders and attention mechanisms for drug-target interaction prediction, optimized via supervised learning without domain adaptation or meta-learning modules.
BioBridge$_{CDAN}$	Leverages Conditional Domain Adversarial Networks (CDAN) for domain-invariant feature alignment, focusing on generic domain shifts without explicit category awareness.
BioBridge$_{CADA}$	Enhances cross-domain generalization using Category-Aware Domain Adversarial Learning (CADA) to align feature distributions between known and unseen protein families, distinguishing positive/negative interactions during adversarial training.
BioBridge$_{Meta}$	Integrates dynamic prototype meta-learning to associate weakly annotated interactions, enabling few-shot predictions via task-specific prototypes and adaptive focal loss.
BioBridge$_r$	A regression-focused variant with an affinity decoder to predict continuous binding strengths (e.g., pIC50/Kd), complementing interaction classification with quantitative binding analysis.

S3 Experimental setting

Evaluation strategies and metrics: We evaluate our model's classification accuracy across three public datasets: BindingDB [8, 3], BioSNAP [27], and Human [16], with the test set simulating an unknown real-world scenario. Table S1 outlines dataset characteristics. Considering the bias highlighted by Chen et al. [6], which suggests that models tend to memorize existing drug patterns rather than actual interactions, we primarily assess the model's generalization capabilities on unknown drug-target pairs. We investigate two data partitioning strategies: the cold pair split, which allocates 70% of drug/protein pairs for training and splits the remaining 30% into validation (30%) and test sets (70%), and the cross-domain partitioning, which follows the clustering-based approach of DrugBAN [4]. This cold pair split strategy ensures that all test drugs and proteins are not observed during training so that prediction on test data cannot rely solely on the features of known drugs or proteins. For the latter, drugs and proteins are clustered using the ECFP4 and PSC algorithms, respectively, with 60% of the clusters designated as the source domain and the remaining 40% as the target domain. The cluster-based partitioning ensures that the model does not even see drug-target pairs similar to those in the test set.
In meta-learning contexts, we apply PSC clustering to proteins in BindingDB and BioSNAP datasets, coupled with scaffold splitting for drug molecules. We define tasks based on protein and drug molecule clusters to ensure diverse yet related query and support sets, mirroring the drug discovery scenario with limited novel drug-target pairs but available informative interactions. Tasks with fewer than six instances are consolidated into the source domain, enriching its diversity and preparing for a challenging target domain task. The first 40% of the cumulative distribution clusters are allocated to the source domain, with the remaining 60% forming the target domain, which is further divided into training (70%) and test sets (30%) to mimic real-world distribution variations. Testing incorporates at least twice the number of tasks as the test set size, establishing a stringent training and evaluation framework. Clustering details are detailed in Table S4.
We use AUROC (area under the receiver operating characteristic curve), AUPRC (area under the precision-recall curve), and ACC (Accuracy) as primary metrics for classification tasks. The model with the highest AUROC on the validation set is selected for testing, and its results are reported on the test set. To ensure reliability, we conduct five independent runs with different random seeds.
Implementation: For conventional training, the BioBridge model utilizes the Adam optimizer at a learning rate of 5e-5 with batch sizes of 64 over 100 epochs. In contrast, for meta-learning scenarios, the learning rate is increased to 1e-4, with batch sizes reduced to 32, and the training is conducted for 50 epochs. The best-performing model is selected at each epoch based on the AUROC score achieved on

the validation set, which is then used to evaluate the final performance of the test set. The protein feature stem consists of two 128-dimensional 1D convolutional layers, and the molecule feature stem has two fully connected layers with 128 dimensions. The target feature encoder has three 1D-CNN layers with 128 filters and kernel sizes of 3, 6, and 9, along with max-pooling layers. The drug feature encoder includes three GCN layers with 128 dimensions for feature selection. Max-pooling and GCN layers with 128 dimensions perform feature selection. The maximum sequence length for targets is 1200, and the maximum number of atoms for drug molecules is 290. The bilinear attention module has two heads with a latent embedding size of 768 and an average pooling window size of 3. The gated attention unit has a total hidden layer size of 256, with Query and Key layers at 128. The fully connected decoder has 512 hidden neurons. The implementation of meta-learning is based on learn2learn [2].
Baselines: In summary, we compare our model with seven others for villain DTI prediction: (1) GNN-CPI [23], which employs a graph neural network for drugs and a CNN for proteins, linking latent vectors for interaction prediction; (2) DeepConv-DTI [14], which utilizes a CNN with global max-pooling for protein sequences and a fully connected network for ECFP4 drug fingerprints; (3) GraphDTA [18], which combines a graph neural network for drug molecular graphs with a CNN for protein sequences through simple concatenation; (4) TransformerCPI [6], which enhances interaction prediction using sequence-based deep learning with a self-attention mechanism; (5) MolTrans [10], which applies a Transformer for encoding and a CNN-based module for substructure interactions; and (6) DrugBAN [4], which models DTI using a graph neural network for drug molecular graphs and a CNN for proteins, incorporating a bilinear attention mechanism.
Given the innovative nature of our research, existing meta-learning studies focused on specialized domains and tailored encoders offer limited applicability. Therefore, we compare our model against broader meta-learning approaches that may yield more versatile solutions. In our meta-task experiments, we use BioBridge as the primary network for data encoding, evaluating its performance against five advanced learning strategies designed for rapid adaptation: (1) MAML++ [1], which enhances MAML [7] by introducing an additional task for improved adaptability; (2) ANIL [19], which employs a separate network to predict necessary model adjustments for new tasks; (3) Prototypical Networks [20], which learns from representative examples to quickly grasp new tasks; and (4) MetaOptNet [15], which refines model performance on new tasks by fine-tuning essential hyperparameters.
We follow the recommended hyperparameter settings for each model.

S4 Mathematical notations

Table S3 lists the key notations used in this paper with descriptions.

S5 Meta-learning problem definition

The proposed meta-learning framework identifies shared patterns across tasks and adapts quickly to new tasks, improving predictive accuracy. It is framed as an N_w-way, N_s-shot problem, where N_w is the number of classes, and N_s is the number of labeled examples per class. Each training iteration samples N_w classes to construct a task T. Drug-target pairs are represented as $X = \{x_1, x_2, \ldots, x_n\}$, where $x_i = \{\mathcal{D}_i, \mathcal{P}_i\}$ includes a drug $\mathcal{D}_i$ and its target protein $\mathcal{P}_i$. Corresponding class labels are $Y = \{y_1, y_2, \ldots, y_n\}$. Task T includes a support set $S = \{(X_s, Y_s)\}$ and a query set $Q = \{(X_q, Y_q)\}$, constructed by randomly selecting N_s and N_q samples per class. The goal is to train the model to adapt to the query set Q using the support set S. In drug-target interaction (DTI) prediction, $N_w = 2$.

S6 Meta unseen pair split strategy

For cross-domain meta-learning performance evaluation, we perform clustering on target proteins from the BindingDB and BioSNAP datasets using single-linkage clustering. This method maintains inter-

Table S3: Summary of key notations in the BioBridge Model

Notation	Description
$\mathcal{D}_i \in \mathbb{R}^{L_d \times \Theta_d}$	Drug molecule representation at the i-th stage (L_d: number of atoms, Θ_d: feature dimension).
$\mathcal{P}_i \in \mathbb{R}^{L_p \times \Theta_p}$	Protein representation at the i-th stage (L_p: sequence length, Θ_p: feature dimension).
$\mathcal{I}_i \in \mathbb{R}^{N \times M}$	Drug-protein interaction matrix at the i-th stage (N: protein substructures, M: drug atoms).
$\mathcal{O} \in \mathbb{R}^{dim}$	Integrated interaction pattern representation (dim: output dimension).
f^b, f^d, f^p	Binding interaction encoder, drug encoder, and protein encoder.
$\mathcal{L}_s, \mathcal{L}_d, \mathcal{L}_f$	Source domain cross-entropy loss, adversarial loss, and Focal Loss.
$R_s, R_t \in \mathbb{R}^{dim}$	Source and target domain features after Gradient Reversal Layer (GRL).
D_0, D_1	Adversarial discriminators for domain classification (negative/positive).
$\mathcal{T} = \{T_1, \ldots, T_N\}$	Meta-task set, where each task T_i contains a support set S_i and query set Q_i.
$S_i = \{(X_s, Y_s)\}$	Support set: labeled drug-target pairs (2-way k-shot).
$Q_i = \{(X_q, Y_q)\}$	Query set: novel drug-target pairs to predict.
$\mathbf{P}[n, q, c] \in \mathbb{R}^d$	Dynamic prototype for class c in task n, query q.
$\mathbf{O}_s \in \mathbb{R}^{N \times 2k \times d}$	Support set interaction features (N: tasks, k: shots per class, d: feature dim).
$\mathbf{O}_q \in \mathbb{R}^{N \times k_q \times d}$	Query set interaction features (k_q: query samples per task).
$\mathbf{Q}, \mathbf{K}, \mathbf{V} \in \mathbb{R}^{d_h}$	Query, Key, Value representations in dynamic prototype learning.
$s_{nqc} = \cos(\mathbf{P}[n, q, c], \mathbf{X}_q)$	Cosine similarity between query and prototype.
p_{nq}	Classification probability of query samples (via softmax on s_{nqc}).
$\gamma_1, \gamma_2, \beta_1, \beta_2$	Scaling factors and biases in dynamic prototype learning.
α, γ, λ	Focal Loss class weight, modulation factor, and adversarial loss weight.

cluster distances above a threshold λ. Furthermore, molecular scaffold-based clustering is applied to the molecules within these datasets. This method fosters a cross-domain environment by avoiding cluster convergence and preserving intra-cluster homogeneity, mirroring the drug discovery process where existing drug-target interactions inform the prediction of new ones.
We utilize integral PSC features to represent target proteins and measure pairwise distances using Jaccard distance for ECFP4 and cosine distance for PSC. We set $\lambda = 0.5$ in protein clustering to prevent large cluster formation and ensure sample separation. Table S4 presents the sample counts of the ten most significant clusters, revealing that BindingDB has a more uniform cluster distribution than BioSNAP in drug clustering. Additionally, protein clustering tends to form many small clusters across both datasets, indicating a lower average similarity among proteins compared to drugs.

S7 Scalability and computational efficiency

Designed for scalability, BioBridge achieves optimal performance through the integration of its components. As shown in Figure S1, BioBrigde demands minimal training resources, fitting well within 60 epochs on an RTX4090, and can be expedited with early stopping. At a batch size of 64, the vanilla BioBridge completes an epoch in 1.53 minutes using 9.2G of VRAM. BioBridge$_{CADA}$ takes 2.5 minutes per epoch

Table S4: Size of the ten largest clusters in the BindingDB and BioSNAP datasets generated by the meta unseen pair split.

Dataset	Object	#1	#2	#3	#4	#5	#6	#7	#8	#9	#10
BindingDB	Drug	175	116	105	90	82	75	74	63	56	53
BioSNAP	Drug	475	353	59	56	37	32	29	26	23	22
BindingDB	Protein	17	15	15	12	10	10	10	9	9	8
BioSNAP	Protein	8	8	8	6	5	4	4	4	4	4

with 13.7G VRAM, while BioBridge$_{Meta}$ requires just 35 seconds per epoch with 2.1G VRAM.

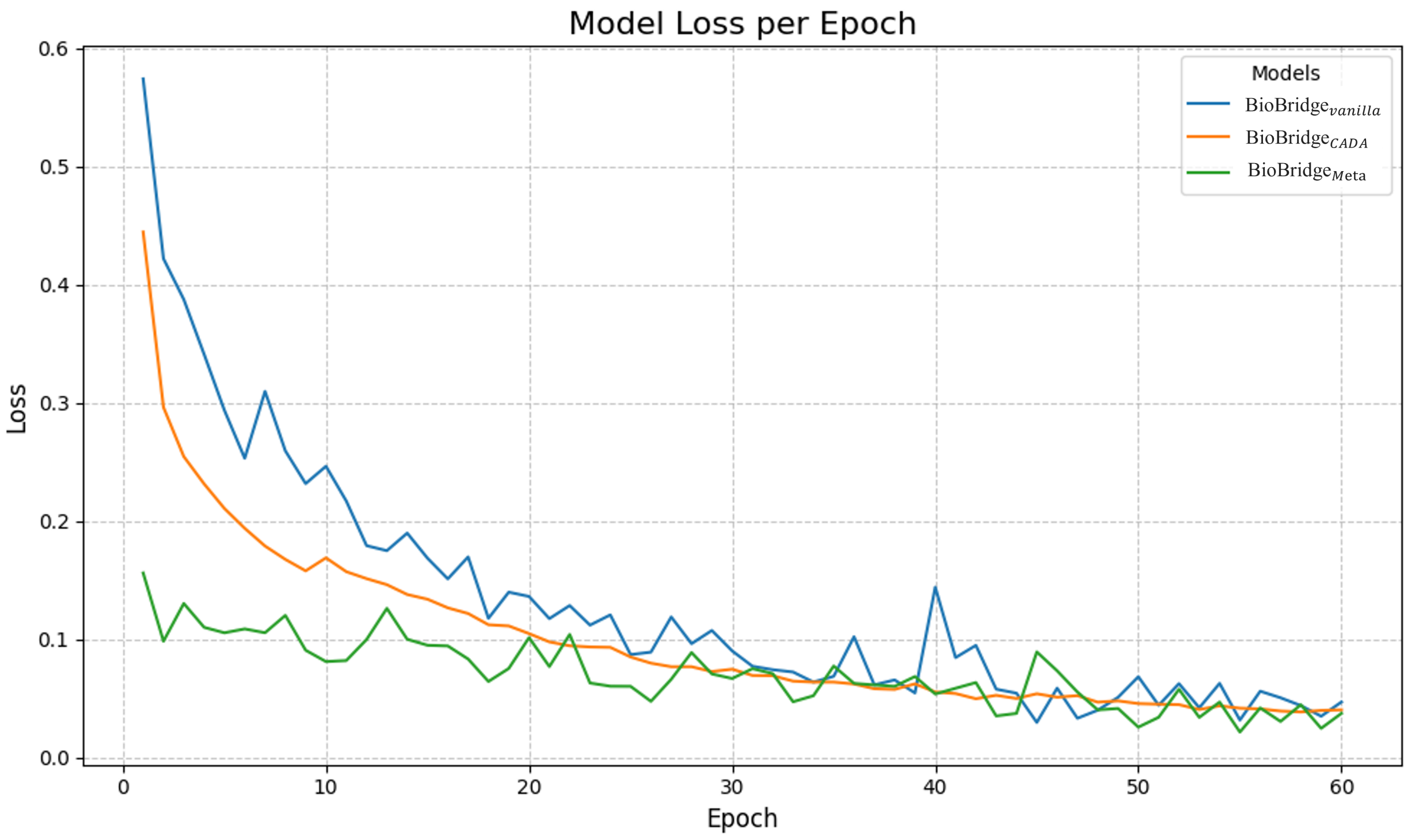

Figure S1: The training losses of the different variants of BioBridge vary with epoch.

S8 Comparison on random and cold-pair split

Table S5 compares the performance of BioBridge on random versus cold splits. Unlike on cold splits, BioBridge's advantage on random splits is less pronounced, suggesting that its multi-level interaction patterns are better suited for unknown drug target pairs while still adequately addressing known pairs.

S9 Compared with the structure-based protein ligand model

We compare BioBridge against structure-based protein-ligand models and find it to be the best among models that do not require 3D crystal structures, matching the performance of some structure-based models. The results in Table S6 suggest that BioBridge can be used cost-effectively for accurate drug-target binding affinity predictions and provides interpretable binding site insights.

Table S5: Performance comparison on the BindingDB, BioSNAP and Human datasets(**Best**, Second Best). Data are expressed as mean ± standard deviation.

Metrics	AUROC	AUPRC	ACC	AUROC	AUPRC	ACC
Datasets	BindingDB			BindingDB(Cold)		
GNN-CPI [23]	0.9418 ± 0.0051*	0.9210 ± 0.0113*	0.8823 ± 0.0021*	0.5893 ± 0.0101*	0.5562 ± 0.0140*	0.5481 ± 0.0182*
DeepConv-DTI [14]	0.9502 ± 0.0030*	0.9308 ± 0.0042*	0.8924 ± 0.0027*	0.6204 ± 0.0128*	0.5913 ± 0.0154*	0.5879 ± 0.0251*
GraphDTA [18]	0.9517 ± 0.0009*	0.9371 ± 0.0009*	0.8989 ± 0.0023*	0.6191 ± 0.0142*	0.6006 ± 0.0201	0.6003 ± 0.0205*
TransformerCPI [6]	0.9511 ± 0.0012*	0.9489 ± 0.0017*	0.8954 ± 0.0011*	0.6559 ± 0.0124*	0.5941 ± 0.0132*	0.6096 ± 0.0131*
HyperAttDTI [25]	0.9593 ± 0.0017*	0.9513 ± 0.0018*	0.9012 ± 0.0028*	0.6612 ± 0.0161	0.5988 ± 0.0213	0.6332 ± 0.0226*
MolTrans [10]	0.9541 ± 0.0022*	0.9414 ± 0.0009*	0.9023 ± 0.0012*	0.5948 ± 0.0144*	0.5225 ± 0.0176*	0.5324 ± 0.0148*
DrugBAN [4]	0.9624 ± 0.0009*	0.9512 ± 0.0009*	0.9064 ± 0.0041*	0.6553 ± 0.0187*	0.6004 ± 0.0151	0.6121 ± 0.0212*
BioBridge$_{vanilla}$	**0.9662 ± 0.0009**	**0.9543 ± 0.0009**	**0.9143 ± 0.0032**	**0.6801 ± 0.0151**	**0.6269 ± 0.0197**	**0.6341 ± 0.0271**
Datasets	BioSNAP			BioSANP(Cold)		
GNN-CPI [23]	0.8832 ± 0.0052*	0.8913 ± 0.0042*	0.8171 ± 0.0024*	0.6331 ± 0.0153*	0.6574 ± 0.0112*	0.6162 ± 0.0091*
DeepConv-DTI [14]	0.8900 ± 0.0041*	0.8954 ± 0.0036*	0.8152 ± 0.0053*	0.6534 ± 0.0104*	0.6683 ± 0.0121*	0.6187 ± 0.0136*
GraphDTA [18]	0.8901 ± 0.0082*	0.8921 ± 0.0071*	0.8103 ± 0.0067*	0.6431 ± 0.0112*	0.6730 ± 0.0231*	0.6315 ± 0.0093*
TransformerCPI [6]	0.8945 ± 0.0016*	0.8931 ± 0.0033*	0.8225 ± 0.0045*	0.6803 ± 0.0099*	0.7083 ± 0.0079*	0.6483 ± 0.0119
HyperAttDTI [25]	0.9042 ± 0.0029*	0.9087 ± 0.0039*	0.8312 ± 0.0040*	0.7148 ± 0.0182	**0.7312 ± 0.0150**	0.6636 ± 0.0172
MolTrans [10]	0.9019 ± 0.0037*	0.9044 ± 0.0047*	0.8254 ± 0.0102*	0.6724 ± 0.0146*	0.6968 ± 0.0104*	0.6402 ± 0.0118
DrugBAN [4]	0.9084 ± 0.0022*	0.9119 ± 0.0041*	0.8339 ± 0.0083*	0.6589 ± 0.0191*	0.6673 ± 0.0119*	0.6324 ± 0.0122*
BioBridge$_{vanilla}$	**0.9161 ± 0.0021**	**0.9192 ± 0.0031**	**0.8428 ± 0.0018**	**0.7161 ± 0.0110**	0.7231 ± 0.0108	**0.6648 ± 0.0187**
Datasets	Human			Human(Cold)		
GNN-CPI [23]	0.9791 ± 0.0034*	0.9807 ± 0.0031	0.9191 ± 0.0100	0.8143 ± 0.0231*	0.7514 ± 0.0308	0.7047 ± 0.0311*
DeepConv-DTI [14]	0.9802 ± 0.0021*	0.9813 ± 0.0013	0.9200 ± 0.0037	0.8317 ± 0.0452*	0.7856 ± 0.0524	0.7281 ± 0.0182*
GraphDTA [18]	0.9809 ± 0.0012*	**0.9822 ± 0.0009**	0.9242 ± 0.0056	0.8301 ± 0.0391	0.7723 ± 0.0463	0.7192 ± 0.0353
TransformerCPI [6]	0.9730 ± 0.0011*	0.9756 ± 0.0021*	0.9358 ± 0.0101	0.8382 ± 0.0233*	0.7712 ± 0.0270	0.7314 ± 0.0196*
HyperAttDTI [25]	**0.9842 ± 0.0011**	0.9812 ± 0.0029	0.9354 ± 0.0071	0.8489 ± 0.0321	0.7870 ± 0.0271	0.7563 ± 0.0287
MolTrans [10]	0.9798 ± 0.0022*	0.9791 ± 0.0018	0.9322 ± 0.0119	0.8342 ± 0.0257*	0.7821 ± 0.0384	0.7520 ± 0.0194
DrugBAN [4]	0.9823 ± 0.0021	0.9819 ± 0.0031	0.9334 ± 0.0088	0.8511 ± 0.0276	0.7894 ± 0.0427	0.7574 ± 0.0311
BioBridge$_{vanilla}$	0.9842 ± 0.0031	0.9822 ± 0.0027	**0.9359 ± 0.0102**	**0.8772 ± 0.0341**	**0.7951 ± 0.0582**	**0.7618 ± 0.0240**

∗ Significantly different ($p < 0.05$) from the corresponding BioBridge metric value; one-way analysis of variance (ANOVA).

Table S6: Performance comparison on the PDB2020 datasets. (**Best Sequence-based**, Second Best Sequence-based). Data are expressed as mean ± standard deviation.

Methods	Hi-res	Co-crystal	PDBBind v2020					
	Structure	Complex	RMSE ↓	MAE ↓	Pearson ↑	Spearman ↑	r_m^2 ↑	CI ↑
Pafnucy [21]	✓	✓	1.4353 ± 0.0182	1.1447 ± 0.0189*	0.6359 ± 0.0083*	0.5872 ± 0.0089*	0.3487 ± 0.0162	0.7074 ± 0.0041*
OnionNet [26]	✓	✓	1.4039 ± 0.0124	1.1038 ± 0.0147	0.6483 ± 0.0079	0.6024 ± 0.0132*	0.3813 ± 0.0119*	0.7178 ± 0.0053*
IGN [11]	✓	✓	1.4047 ± 0.0253	1.1169 ± 0.0304	0.6623 ± 0.0137	0.6389 ± 0.0214	0.3854 ± 0.0209*	0.7309 ± 0.0094
SMINA [13]	✓	✗	1.4662 ± 0.0089*	1.1613 ± 0.0074*	0.6658 ± 0.0057	0.6639 ± 0.0192*	0.3917 ± 0.0313	0.7408 ± 0.0082
GNINA [17]	✓	✗	1.7409 ± 0.0143*	1.4137 ± 0.0152*	0.4958 ± 0.0119*	0.4947 ± 0.0113*	0.2094 ± 0.0098*	0.6749 ± 0.0047*
dMaSIF [22]	✓	✗	1.4509 ± 0.0327	1.1364 ± 0.0319	0.6298 ± 0.0183*	0.5889 ± 0.0412	0.3479 ± 0.0297	0.7107 ± 0.0173
GraphDTA [18]	✗	✗	1.5640 ± 0.0630*	1.2230 ± 0.0660*	0.6120 ± 0.0160*	0.5700 ± 0.0500*	0.3060 ± 0.0390*	0.7030 ± 0.0190*
TransformerCPI [6]	✗	✗	1.4930 ± 0.0500*	1.2010 ± 0.0370*	0.6040 ± 0.0240*	0.5510 ± 0.0290*	0.2550 ± 0.0270*	0.6770 ± 0.0110*
MolTrans [10]	✗	✗	1.5990 ± 0.0600*	1.2710 ± 0.0510*	0.5390 ± 0.0570*	0.4740 ± 0.0520*	0.2420 ± 0.0450*	0.6660 ± 0.0200*
DrugBAN [4]	✗	✗	1.4800 ± 0.0460*	1.1590 ± 0.0450*	0.6570 ± 0.0180	0.6120 ± 0.0270*	0.3190 ± 0.0210*	0.7200 ± 0.0110
BioBridge$_{vanilla}$	✗	✗	**1.4172 ± 0.0012**	**1.1038 ± 0.0139**	**0.6636 ± 0.0090**	**0.6331 ± 0.0170**	**0.3543 ± 0.0180**	**0.7285 ± 0.0090**

∗ Significantly different ($p < 0.05$) from the corresponding BioBridge metric value; one-way analysis of variance (ANOVA).

S10 Protein Variability as a Performance Bottleneck

Evaluations on cross-domain scenariosnovel drugs (Meta Unseen Drug) and proteins (Meta Unseen Protein)reveal significantly greater prediction challenges for unseen proteins, with a 20% performance gap compared to novel drugs (Figure S2), highlighting protein variability as a key bottleneck in drug-target interaction prediction.

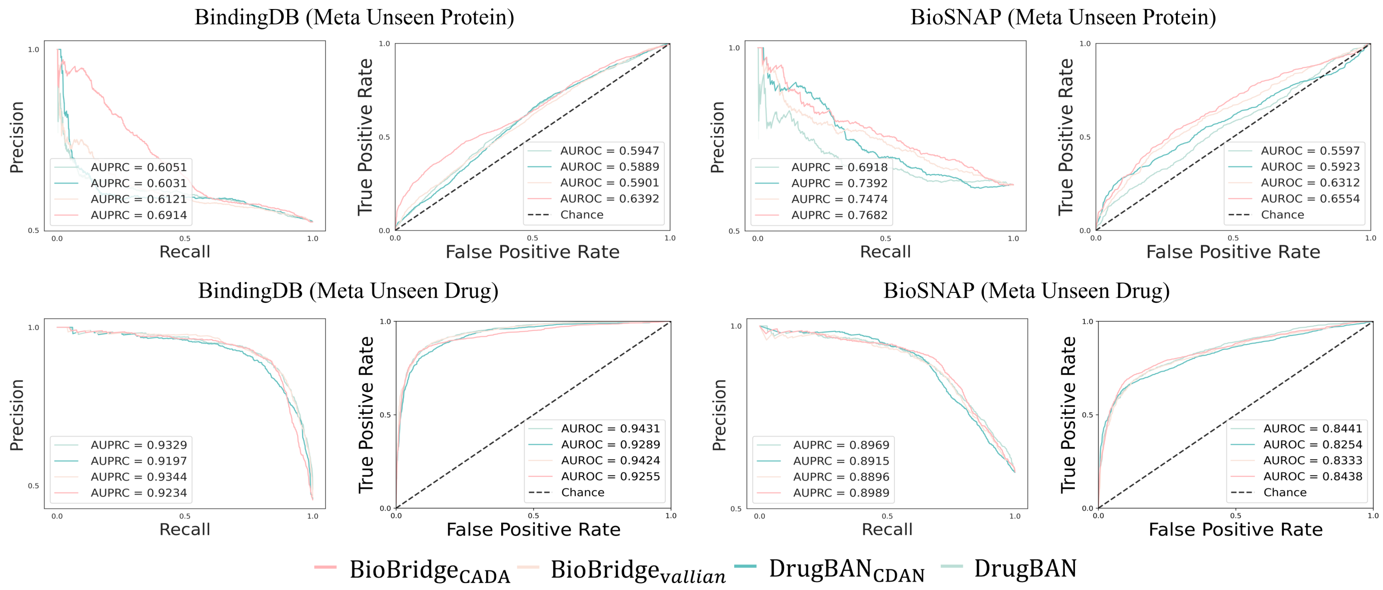

Figure S2: Cross-domain prediction performance comparison between novel drugs (Meta Unseen Drug) and proteins (Meta Unseen Protein).

S11 Comparison on specific meta unseen protein split

Under the specific meta-unseen protein split paradigm, each meta-task's support and query sets comprise binding profiles from the same target with distinct drugs. This stringent meta-learning strategy necessitates predicting new interactions with consistent drug-target pairs. The results are shown in Table S7.

Table S7: Few shot comparison of specific meta unseen protein splitting on the BindingDB and BinSNAP datasets (**Best**, Second Best). Data are expressed as mean ± standard deviation.

Dataset	BindingDB(Specific Meta Unseen Protein)			BioSNAP(Specific Meta Unseen Protein)		
Metric	AUROC	AUPRC	ACC	AUROC	AUPRC	ACC
Setting	1-shot					
MAML++ [1]	0.6477 ± 0.0133*	0.6343 ± 0.0164*	0.6061 ± 0.0093*	0.5804 ± 0.0108*	0.5705 ± 0.0125*	0.5565 ± 0.0082*
Protypes [20]	0.7186 ± 0.0129*	0.7209 ± 0.0154*	0.6500 ± 0.0087*	0.6601 ± 0.0049*	0.6543 ± 0.0055*	0.6113 ± 0.0067*
MetaOptNet [15]	0.7344 ± 0.0062*	0.7191 ± 0.0063*	0.6711 ± 0.0036*	0.6683 ± 0.0097*	0.6469 ± 0.0099*	0.6248 ± 0.0046
ANIL [19]	0.6951 ± 0.0247*	0.6918 ± 0.0259*	0.6374 ± 0.0181*	0.6560 ± 0.0280*	0.6548 ± 0.0214	0.6051 ± 0.0239
BioBridge$_{Meta}$	**0.7824 ± 0.0047**	**0.7893 ± 0.0050**	**0.6960 ± 0.0027**	**0.6861 ± 0.0128**	**0.6841 ± 0.0139**	**0.6286 ± 0.0091**
Setting	3-shot					
MAML++ [1]	0.7831 ± 0.0156*	0.7806 ± 0.0160*	0.7079 ± 0.0152*	0.6783 ± 0.0026*	0.6723 ± 0.0017*	0.6257 ± 0.0032*
Protypes [20]	0.7582 ± 0.0207*	0.7588 ± 0.0208*	0.6849 ± 0.0195*	0.7160 ± 0.0107*	0.7103 ± 0.0111*	0.6541 ± 0.0032*
MetaOptNet [15]	0.8311 ± 0.0070*	0.8114 ± 0.0048*	0.7604 ± 0.0083	0.7568 ± 0.0077*	0.7341 ± 0.0061*	0.6967 ± 0.0055*
ANIL [19]	0.7768 ± 0.0077*	0.7742 ± 0.0078*	0.7008 ± 0.0053*	0.7141 ± 0.0314*	0.7058 ± 0.0356*	0.6581 ± 0.0193*
BioBridge$_{Meta}$	**0.8621 ± 0.0023**	**0.8663 ± 0.0019**	**0.7693 ± 0.0028**	**0.7919 ± 0.0072**	**0.7910 ± 0.0084**	**0.7130 ± 0.0056**
Setting	5-shot					
MAML++ [1]	0.8190 ± 0.0410	0.8173 ± 0.0450*	0.7390 ± 0.0305*	0.7190 ± 0.0382*	0.7077 ± 0.0461*	0.6614 ± 0.0273*
Protypes [20]	0.7895 ± 0.0020*	0.7886 ± 0.0003*	0.7105 ± 0.0038*	0.7393 ± 0.0239*	0.7330 ± 0.0259*	0.6730 ± 0.0173*
MetaOptNet [15]	0.8561 ± 0.0142*	0.8388 ± 0.0162*	0.7834 ± 0.0128*	0.7748 ± 0.0010*	0.7537 ± 0.0059*	0.7143 ± 0.0049*
ANIL [19]	0.8079 ± 0.0016*	0.8079 ± 0.0030*	0.7258 ± 0.0043*	0.7394 ± 0.0098*	0.7367 ± 0.0083*	0.6708 ± 0.0052*
BioBridge$_{Meta}$	**0.8802 ± 0.0025**	**0.8828 ± 0.0030**	**0.7894 ± 0.0026**	**0.8249 ± 0.0149**	**0.8254 ± 0.0145**	**0.7446 ± 0.0126**

∗ Significantly different ($p < 0.05$) from the corresponding BioBridge metric value; one-way analysis of variance (ANOVA).

S12 Detailed SPR Experimental Procedures and Results

Surface plasmon resonance (SPR) binding assays were carried out at 25 °C on a Biacore 1K instrument (Cytiva) using CM5 sensor chips to validate two top-ranked BioBridge predictions (Nitraconazole nitrate and methotrexate binding to AA1R). Prior to analysis, all system lines were primed with running buffer ($1\times$ PBS-P$^+$, pH 7.4, supplemented with 5% (v/v) DMSO) to stabilize the baseline. For chip preparation, both flow cells were activated by injecting 0.2 M EDC/0.05 M NHS (1:1 v/v) at 10 μL/min for 420 s. Recombinant human AA1R (50 μg/mL in 10 mM sodium acetate, pH 5.0) was immobilized onto the active cell (FC2) at 10 μL/min for 420 s to achieve $\sim$ 10,000 RU, followed by blocking with 1 M ethanolamine-HCl (pH 8.5) at 10 μL/min for 420 s. The reference cell (FC1) was activated and blocked under identical conditions but with sodium acetate buffer in place of protein. To correct for bulk refractive-index changes caused by DMSO, a solvent calibration series (4.5-5.8% DMSO in running buffer) was prepared by mixing 4.5% and 5.8% DMSO stock solutions (see Table S8) and injected over both flow cells prior to analyte injections; calibration curves were generated and applied during data processing. Nitraconazole nitrate and methotrexate were dissolved in DMSO to 10 mM stocks and serially diluted (two-fold) in running buffer to yield concentrations spanning 0.1-100 μM. Each analyte concentration was injected in triplicate at 10 μL/min for a 150 s association phase, followed by a 300 s dissociation phase in running buffer; surfaces were regenerated between injections with 10 mM glycine-HCl (pH 2.0) at 10 μL/min for 300 s. Sensorgrams from the active cell were double-referenced by subtracting response from the reference cell and blank (running buffer) injections, then fitted globally to a 1:1 Langmuir binding model using Biacore Insight (v. 2.0, Cytiva).
For Nitraconazole nitrate, the global fit yielded.

$$k_a = 5.46 \times 10^4\,\mathrm{M^{-1}s^{-1}}, \quad k_d = 1.22 \times 10^{-1}\,\mathrm{s^{-1}}, \quad K_D = 2.23 \times 10^{-6}\,\mathrm{M};$$

for methotrexate,

$$k_a = 1.09 \times 10^4\,\mathrm{M^{-1}s^{-1}}, \quad k_d = 5.95 \times 10^{-3}\,\mathrm{s^{-1}}, \quad K_D = 5.48 \times 10^{-7}\,\mathrm{M}.$$

These micromolar-to-submicromolar affinities confirm BioBridge's ability to prioritize novel high-confidence drug-target pairs under cold-start conditions.

Table S8: Preparation of DMSO Solvent Calibration Solutions.

**Vial**	1	2	3	4	5	6	7	8
**4.5% DMSO (μL)**	0	200	400	600	800	1000	1200	1400
**5.8% DMSO (μL)**	1400	1200	1000	800	600	400	200	0

S13 Module ablation experiments

The study presented in Table S9 evaluates the effectiveness of the Multi-scale Awareness, Stem, and GAU components within the BioBridge multi-level encoder. The removal of these elements demonstrated their contributions to the overall performance, with BioBridge consistently outperforming other models across various metrics. This underscores the importance of multi-level feature models in facilitating knowledge transfer, as they outshine their single-level counterparts.
Figure S3 further examines the CADA module's performance in cross-domain tasks. It reveals that BioBridge achieved the highest results when equipped with CADA. This is followed by DrugBAN with CADA and then BioBridge with CDAN. These findings suggest that a multi-level approach, combined with an understanding of biological categories, is crucial for accurately capturing drug-target interactions.

S14 BioBridge details

As depicted in Figure S4, the design details of BioBridge encompass a multi-level encoder along with its constituent modules and a dynamic prototype algorithm for cross-domain meta-learning.

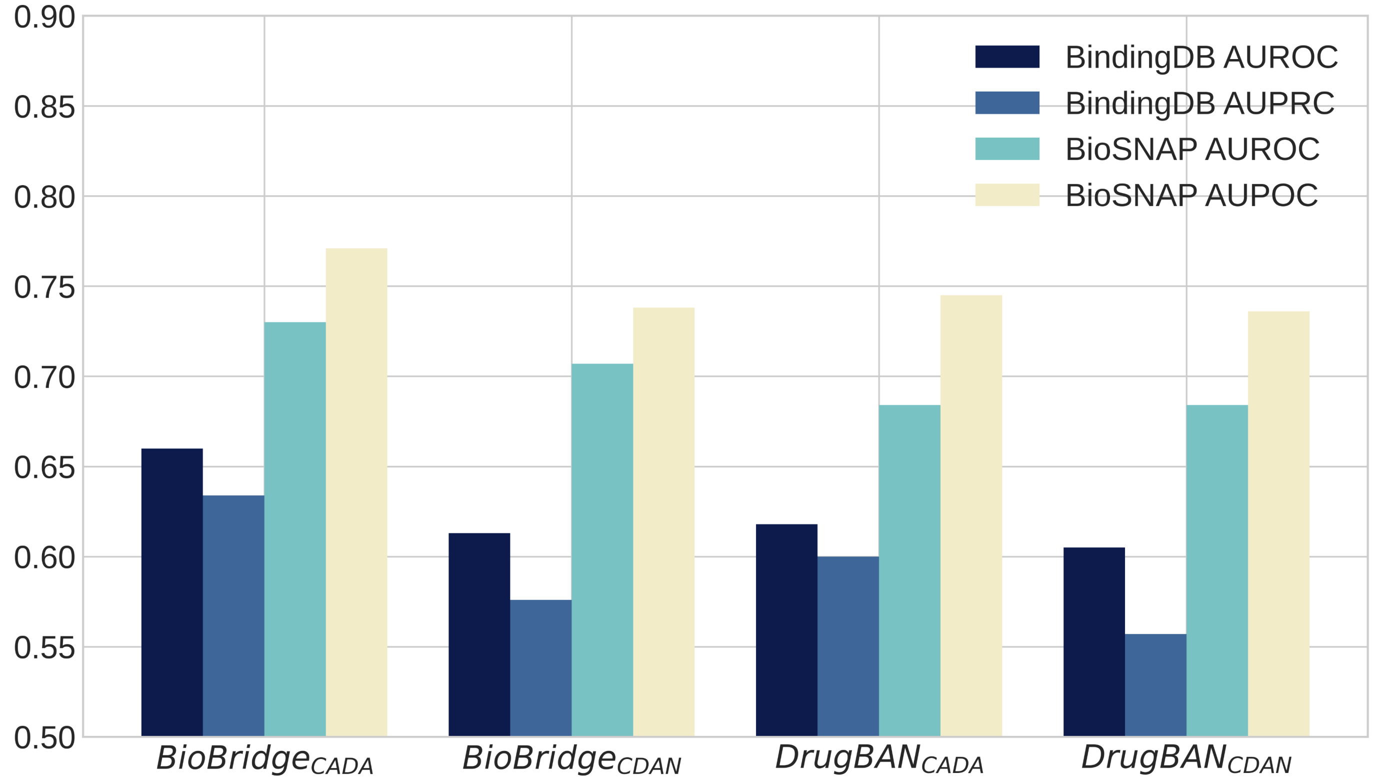

Figure S3: Ablation experiments for CADA modules in cross-domain settings.

Table S9: Ablation experiments of BioBridge encoders on BindingDB and BioSNAP datasets (**Best**, Second Best). Data are expressed as mean ± standard deviation.

MA	Stem	GAU	AUROC	AUPRC	ACC	AUROC	AUPRC	ACC
	Datasets			BindingDB (Cold)			BioSNAP (Cold)	
✓	✓	✓	**0.6799 ± 0.0153**	**0.6271 ± 0.0197**	**0.6339 ± 0.0275**	0.7160 ± 0.0109	**0.7233 ± 0.0098**	**0.6648 ± 0.0187**
✗	✓	✓	0.6609 ± 0.0121	0.6077 ± 0.0026	0.5989 ± 0.0180	0.6896 ± 0.0068	0.6912 ± 0.0102	0.6262 ± 0.0102
✓	✗	✓	0.6336 ± 0.0126	0.5612 ± 0.038	0.5862 ± 0.0352	0.6863 ± 0.0510	0.6927 ± 0.0683	0.6436 ± 0.0314
✗	✗	✓	0.6767 ± 0.0158	0.6203 ± 0.0061	0.6267 ± 0.0286	0.6886 ± 0.0239	0.6916 ± 0.0256	0.6436 ± 0.0216
✓	✗	✗	0.6648 ± 0.0023	0.6022 ± 0.0182	0.6267 ± 0.0083	**0.7189 ± 0.0092**	0.7173 ± 0.0126	0.6637 ± 0.0218
✗	✗	✗	0.6244 ± 0.0215	0.5802 ± 0.0164	0.5714 ± 0.0452	0.7112 ± 0.0243	0.7181 ± 0.0192	0.6639 ± 0.0165
	SADTI		0.6558 ± 0.0122	0.6002 ± 0.0145	0.6123 ± 0.0232	0.6619 ± 0.0100	0.6102 ± 0.0145	0.6223 ± 0.0232
	Datasets			BindingDB (Random)			BioSNAP (Random)	
✓	✓	✓	**0.9660 ± 0.0009**	**0.9543 ± 0.0009**	**0.9143 ± 0.0032**	**0.9161 ± 0.0021**	**0.9192 ± 0.0031**	**0.8428 ± 0.0018**
✗	✓	✓	0.9637 ± 0.0022	0.9512 ± 0.0030	0.9091 ± 0.0007	0.9124 ± 0.0021	0.9152 ± 0.0035	0.8420 ± 0.0006
✓	✗	✓	0.9636 ± 0.0014	0.9516 ± 0.0014	0.9123 ± 0.0016	0.9127 ± 0.0020	0.9174 ± 0.0029	0.8381 ± 0.0048
✗	✗	✓	0.9625 ± 0.0013	0.9500 ± 0.0024	0.9031 ± 0.0013	0.9105 ± 0.0030	0.9121 ± 0.0039	0.8368 ± 0.0050
✓	✗	✗	0.9632 ± 0.0006	0.9498 ± 0.0039	0.9086 ± 0.0015	0.9093 ± 0.0019	0.9118 ± 0.0041	0.8391 ± 0.0046
✗	✗	✗	0.9618 ± 0.0030	0.9508 ± 0.0035	0.9050 ± 0.0090	0.9090 ± 0.0044	0.9105 ± 0.0053	0.8374 ± 0.0068
	SADTI		0.9624 ± 0.0009	0.9512 ± 0.0009	0.9064 ± 0.0041	0.9081 ± 0.0032	0.9109 ± 0.0039	0.8329 ± 0.0063

The BioBridge encoder (Figure S4(a)) models the interaction forces between protein sequences and molecular graphs. Inspired by human-like multi-level understanding and the fact that features from shallow network layers are more readily transferable across domains compared to those from deeper layers [5, 24]. Accordingly, BioBridge employs a multi-level aware encoder to capture a broader range of binding patterns. BioBridge enhances protein and molecule embeddings through a series of Stem layers, followed by a multi-layer extraction layer paired with a bilinear attention network to identify local drug-target interactions. A gated attention unit then integrates these interaction forces, enabling the model to predict

the likelihood or affinity of protein-ligand interactions with a simple decoder.

Input and Stem: The input consists of target protein FASTA sequences and drug molecular graphs. These are processed through embedding layers to generate residue features $\mathcal{P}^{emb} \in \mathbb{R}^{\mathcal{L}_p \times \Theta_t}$ for proteins and atomic features $\mathcal{D}^{emb} \in \mathbb{R}^{\mathcal{L}_d \times \Theta_d}$ for drugs, where $\mathcal{L}_p$ and $\mathcal{L}_d$ represent the protein sequence length and the number of atoms, respectively, and Θ_t and Θ_d are the feature dimensions for proteins and atoms. To enhance structural information, we apply 1D convolutional layers to refine $\mathcal{P}^{emb}$, yielding $\mathcal{P}^{stem} \in \mathbb{R}^{\mathcal{L}_p \times \Theta_t}$, while drug graph features $\mathcal{D}^{emb}$ are processed through fully connected layers to produce $\mathcal{D}^{stem} \in \mathbb{R}^{\mathcal{L}_d \times \Theta_d}$.

Structure Extractor: The Protein Structural Extractor consists of three CNN blocks that generate multi-level features from the protein sequence. Each block treats the sequence as overlapping trimers (e.g., MRIDKS... GKAQ' → MRI', RID,' IDK,'...), using a 3×3 kernel, BatchNorm, MaxPool, and 1×1 convolutions to refine residue features.

The Drug Structural Extractor uses three GCN blocks to aggregate features from bonded atoms [12], processing through GCN layers, max-pooling, and additional GCN layers for selecting local molecular interactions:

$$
\begin{aligned}
\mathcal{D}_i^{ex} &= BN_i^{ex}(ReLu(GCN_i^{ex}(\mathcal{D}_{i-1}^{ex}))) \\
\mathcal{D}_i^{out} &= GCN_i^{out}(Maxpool_i^{out}(\mathcal{D}_i^{ex})) \\
\mathcal{P}_i^{ex} &= BN_i^{ex}(ReLu(CNN_i^{ex}(\mathcal{P}_{i-1}^{ex}))) \\
\mathcal{P}_i^{out} &= CNN_i^{out}(Maxpool_i^{out}(\mathcal{P}_i^{ex}))
\end{aligned} \tag{1}
$$

This is done for $i = 1, 2, 3$, with $\mathcal{D}_0^{ex} = \mathcal{D}^{stem}$ and $\mathcal{P}_0^{ex} = \mathcal{P}^{stem}$. Here, $\mathcal{P}_i^{ex}$ and $\mathcal{D}_i^{ex}$ are the extracted features for trimeric residues and drug atoms, respectively. In contrast, $\mathcal{P}_i^{out}$ and $\mathcal{D}_i^{out}$ represent the selected features at each level of the model.

Bilinear Attention Network: To capture local interactions between drugs and proteins, we introduce bilinear attention modules within the same hierarchical model. These modules assign attention weights to each drug-protein interaction and pool these weights into feature vectors (Figure S4(b)).

At each level, protein and drug molecule features are represented as $\mathcal{P}_i^{out} = p_i^1, p_i^2, \ldots, p_i^N$ and $\mathcal{D}_i^{out} = d_i^1, d_i^2, \ldots, d_i^M$, where N and M are the counts of selected protein structures and drug atoms, respectively. The interaction matrix $\mathbf{I}_i$ of size $N \times M$ is formulated as:

$$
\begin{aligned}
\mathbf{I}_i = &((\mathbf{1} \cdot \mathbf{q_i}^\top) \odot ReLu((\mathcal{D}_i^{out})^\top \mathbf{U}_i)) \cdot \\
&ReLu(\mathbf{V}_i^\top \mathcal{P}_i^{out}) + p_i
\end{aligned} \tag{2}
$$

Here, V_i and U_i are matrices that align protein and drug features into a shared space of dimension dim. We employ the Hadamard product and learn the weights $q \in \mathbb{R}^{k \times dim}$ and bias $p \in \mathbb{R}^{k \times 1}$ to generate k interaction outcomes, emulating a multi-head attention mechanism.

The interaction outcomes are then converted into feature vectors for subsequent tasks, using the interaction matrix I and variables V_i, U_i to obtain $f_i' \in \mathbb{R}^{k \times dim}$. Pooling operations are also used to simplify the features. Here $i \in \{1, 2, 3\}$:

$$
\begin{aligned}
f_i' &= ReLu((\mathcal{D}_i^{out\top} \mathbf{U}_i))^\top \cdot \mathbf{I} \cdot ReLu((\mathcal{P}_i^{out})^\top \mathbf{V_i}) \\
f_i &= \text{AvgPool}(\mathbf{f_i'}, k)
\end{aligned} \tag{3}
$$

This method effectively captures multi-level drug-protein interactions, providing a more diverse set of interaction features.

Gated Attention Unit: We have engineered a streamlined gated unit with attention mechanisms to integrate interaction data across three hierarchical levels, as shown in Figure S4(c).

To integrate interaction data across three hierarchical levels, we design a gated attention unit. Interaction feature vectors $f \in \mathbb{R}^{3 \times dim}$ are projected into a higher-dimensional space $Value \in \mathbb{R}^{3 \times d_h}$ using $W_h \in \mathbb{R}^{dim \times d_h}$. A gate mechanism determines the significance of each feature via learned parameters, and the final feature vector is derived by adjusting the weighted interaction vectors with $W_o \in \mathbb{R}^{d_h \times dim}$:

$$\begin{aligned} &Value = SiLu(W_h f), gate = SiLu(W_g f),\\ &Z = SiLu(W_z f) \\ &Query = Z\gamma_1 + \beta_1, \quad Key = Z\gamma_2 + \beta_2 \end{aligned} \tag{4}$$

Attention coefficients $A \in \mathbb{R}^{3\times 3}$ are calculated as $\frac{1}{3}\text{relu}^2(Query \cdot Key^\intercal)$, and $Value$ is multiplied by A to merge interaction vectors. The final feature vectors $O \in \mathbb{R}^{dim}$ are selected by the *gate* through Hadamard multiplication with the enhanced interaction vectors and adjusted to the desired dimension using $W_o \in \mathbb{R}^{d_h\times dim}$:

$$A = \frac{1}{3}\text{relu}^2(Query \cdot Key^\intercal) \tag{5}$$

$$O = (Value \cdot A \odot gate)W_o \tag{6}$$

This approach enables nuanced representation across different levels, incorporating interaction patterns at all three model levels.

References

[1] Antreas Antoniou, Harrison Edwards, and Amos J. Storkey. How to train your MAML. *CoRR*, abs/1810.09502, 2018.

[2] Sébastien MR Arnold, Praateek Mahajan, Debajyoti Datta, Ian Bunner, and Konstantinos Saitas Zarkias. learn2learn: A library for meta-learning research. *arXiv preprint arXiv:2008.12284*, 2020.

[3] Peizhen Bai, Filip Miljković, Yan Ge, Nigel Greene, Bino John, and Haiping Lu. Hierarchical clustering split for low-bias evaluation of drug-target interaction prediction. In *2021 IEEE International Conference on Bioinformatics and Biomedicine (BIBM)*, pages 641–644. IEEE, 2021.

[4] Peizhen Bai, Filip Miljković, Bino John, and Haiping Lu. Interpretable bilinear attention network with domain adaptation improves drug–target prediction. *Nature Machine Intelligence*, 5(2):126–136, 2023.

[5] Mathilde Caron, Hugo Touvron, Ishan Misra, Hervé Jégou, Julien Mairal, Piotr Bojanowski, and Armand Joulin. Emerging properties in self-supervised vision transformers. In *Proceedings of the IEEE/CVF international conference on computer vision*, pages 9650–9660, 2021.

[6] Lifan Chen, Xiaoqin Tan, Dingyan Wang, Feisheng Zhong, Xiaohong Liu, Tianbiao Yang, Xiaomin Luo, Kaixian Chen, Hualiang Jiang, and Mingyue Zheng. Transformercpi: improving compound–protein interaction prediction by sequence-based deep learning with self-attention mechanism and label reversal experiments. *Bioinformatics*, 36(16):4406–4414, 2020.

[7] Chelsea Finn, Pieter Abbeel, and Sergey Levine. Model-agnostic meta-learning for fast adaptation of deep networks. In *International conference on machine learning*, pages 1126–1135. PMLR, 2017.

[8] Michael K Gilson, Tiqing Liu, Michael Baitaluk, George Nicola, Linda Hwang, and Jenny Chong. Bindingdb in 2015: a public database for medicinal chemistry, computational chemistry and systems pharmacology. *Nucleic acids research*, 44(D1):D1045–D1053, 2016.

[9] Weizhe Hua, Zihang Dai, Hanxiao Liu, and Quoc Le. Transformer quality in linear time. In *International conference on machine learning*, pages 9099–9117. PMLR, 2022.

[10] Kexin Huang, Cao Xiao, Lucas M Glass, and Jimeng Sun. Moltrans: molecular interaction transformer for drug–target interaction prediction. *Bioinformatics*, 37(6):830–836, 2021.

[11] Dejun Jiang, Chang-Yu Hsieh, Zhenxing Wu, Yu Kang, Jike Wang, Ercheng Wang, Ben Liao, Chao Shen, Lei Xu, Jian Wu, et al. Interactiongraphnet: A novel and efficient deep graph representation learning framework for accurate protein–ligand interaction predictions. *Journal of medicinal chemistry*, 64(24):18209–18232, 2021.

[12] Thomas N Kipf and Max Welling. Semi-supervised classification with graph convolutional networks. *arXiv preprint arXiv:1609.02907*, 2016.

[13] David Ryan Koes, Matthew P Baumgartner, and Carlos J Camacho. Lessons learned in empirical scoring with smina from the csar 2011 benchmarking exercise. *Journal of chemical information and modeling*, 53(8):1893–1904, 2013.

[14] Ingoo Lee, Jongsoo Keum, and Hojung Nam. Deepconv-dti: Prediction of drug-target interactions via deep learning with convolution on protein sequences. *PLoS computational biology*, 15(6):e1007129, 2019.

[15] Kwonjoon Lee, Subhransu Maji, Avinash Ravichandran, and Stefano Soatto. Meta-learning with differentiable convex optimization. In *Proceedings of the IEEE/CVF conference on computer vision and pattern recognition*, pages 10657–10665, 2019.

[16] Hui Liu, Jianjiang Sun, Jihong Guan, Jie Zheng, and Shuigeng Zhou. Improving compound–protein interaction prediction by building up highly credible negative samples. *Bioinformatics*, 31(12):i221–i229, 2015.

[17] Andrew T McNutt, Paul Francoeur, Rishal Aggarwal, Tomohide Masuda, Rocco Meli, Matthew Ragoza, Jocelyn Sunseri, and David Ryan Koes. Gnina 1.0: molecular docking with deep learning. *Journal of cheminformatics*, 13(1):43, 2021.

[18] Thin Nguyen, Hang Le, Thomas P Quinn, Tri Nguyen, Thuc Duy Le, and Svetha Venkatesh. Graphdta: predicting drug–target binding affinity with graph neural networks. *Bioinformatics*, 37(8):1140–1147, 2021.

[19] Aniruddh Raghu, Maithra Raghu, Samy Bengio, and Oriol Vinyals. Rapid learning or feature reuse? towards understanding the effectiveness of maml. *arXiv preprint arXiv:1909.09157*, 2019.

[20] Jake Snell, Kevin Swersky, and Richard Zemel. Prototypical networks for few-shot learning. *Advances in neural information processing systems*, 30, 2017.

[21] Marta M Stepniewska-Dziubinska, Piotr Zielenkiewicz, and Pawel Siedlecki. Development and evaluation of a deep learning model for protein–ligand binding affinity prediction. *Bioinformatics*, 34(21):3666–3674, 2018.

[22] Freyr Sverrisson, Jean Feydy, Bruno E Correia, and Michael M Bronstein. Fast end-to-end learning on protein surfaces. In *Proceedings of the IEEE/CVF Conference on Computer Vision and Pattern Recognition*, pages 15272–15281, 2021.

[23] Masashi Tsubaki, Kentaro Tomii, and Jun Sese. Compound–protein interaction prediction with end-to-end learning of neural networks for graphs and sequences. *Bioinformatics*, 35(2):309–318, 2019.

[24] Jason Yosinski, Jeff Clune, Yoshua Bengio, and Hod Lipson. How transferable are features in deep neural networks? *Advances in neural information processing systems*, 27, 2014.

[25] Qichang Zhao, Haochen Zhao, Kai Zheng, and Jianxin Wang. Hyperattentiondti: improving drug–protein interaction prediction by sequence-based deep learning with attention mechanism. *Bioinformatics*, 38(3):655–662, 2022.

[26] Liangzhen Zheng, Jingrong Fan, and Yuguang Mu. Onionnet: a multiple-layer intermolecular-contact-based convolutional neural network for protein–ligand binding affinity prediction. *ACS omega*, 4(14):15956–15965, 2019.

[27] Marinka Zitnik, Rok Sosic, and Jure Leskovec. Biosnap datasets: Stanford biomedical network dataset collection. *Note: http://snap. stanford. edu/biodata Cited by*, 5(1), 2018.

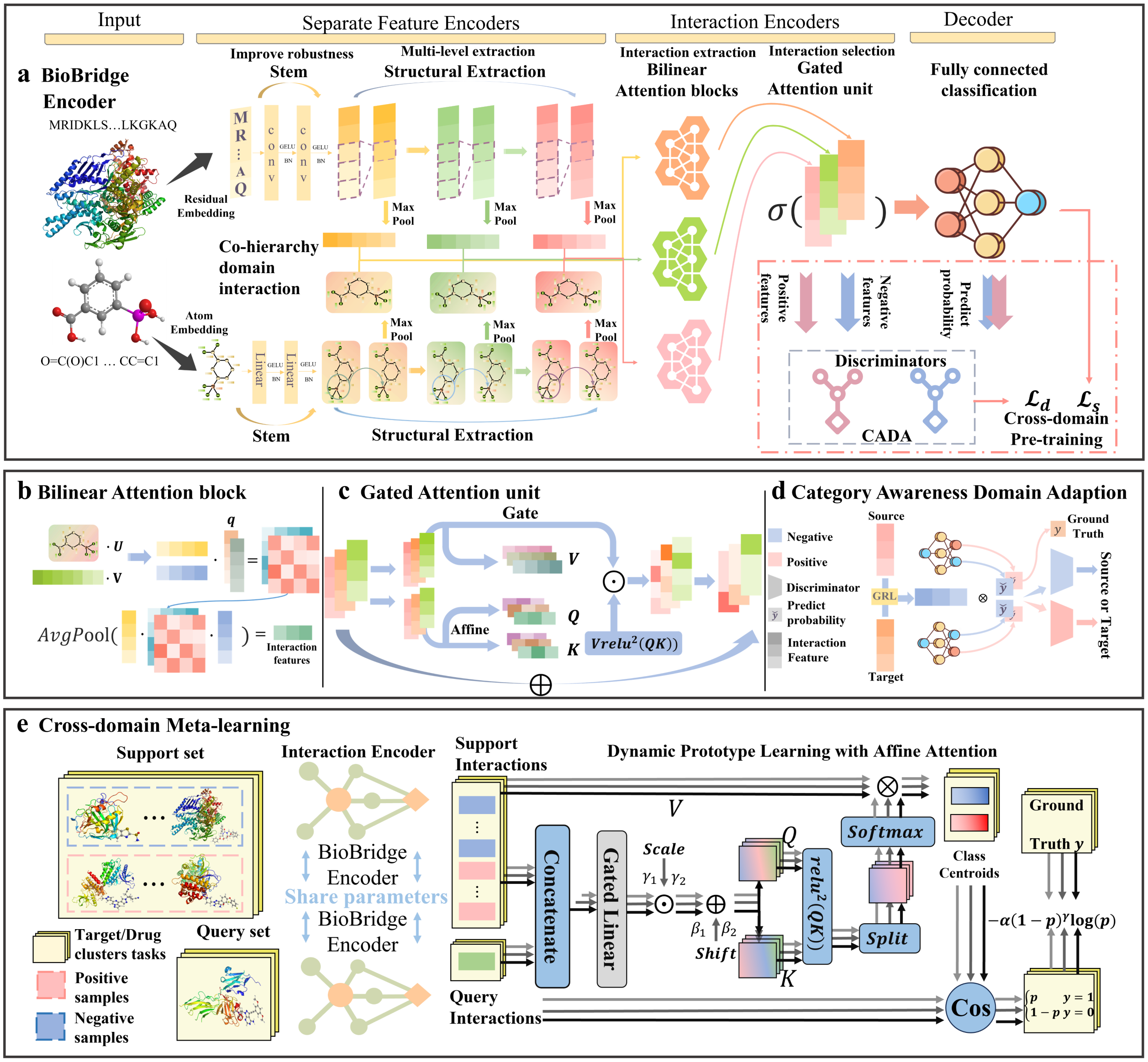

Figure S4: (a) BioBridge encoder processes protein sequences and molecular graphs using CNN and GCN to model their internal force. Bilinear attention captures drug-target interactions, generating diverse interaction fingerprints. These are integrated via gated attention units, with predictions made through a fully connected layer. In cross-domain pre-training, the CADA module enhances generalization through adversarial learning. (b) The bilinear attention network models drug-target interactions by multiplying drug and protein representations with transformation matrices U and V, followed by low-rank bilinear interaction using q. The final interaction representation is obtained through average pooling. (c) The Gated Attention Unit processes input features through a learned gate and affine attention mechanism to produce enhanced output vectors. (d) CADA improves cross-domain generalization by embedding source and target domain representations and class probabilities into a joint representation. Separate discriminators minimize domain classification errors, enhancing domain distinction. (e) Meta-tasks are defined by clustering targets or drugs and dividing them into support and query sets. The BioBridge encoder generates interaction representations for these samples. An affine attention mechanism refines class prototypes by assessing the relative relationships within the support set. Cosine similarity then determines the class of the query set. Focal Loss adaptively weights the learning difficulty of positive and negative samples.
